# Engineering extracellular matrix-based hydrogels for intervertebral disc regeneration

**DOI:** 10.3389/fbioe.2025.1601154

**Published:** 2025-05-01

**Authors:** Mwafaq Kmail, Rusydi Razak, Isma Liza Mohd Isa

**Affiliations:** ^1^ Department of Anatomy, Faculty of Medicine, Universiti Kebangsaan, Kuala Lumpur, Malaysia; ^2^ CÚRAM Research Ireland Centre for Medical Devices, School of Medicine, University of Galway, Galway, Ireland

**Keywords:** intervertebral disc degeneration, lower back pain, hydrogel, extracellular matrix, biomaterials

## Abstract

Lower back pain (LBP) is a major health concern, especially in older adults. A key aetiological factor is intervertebral disc (IVD) degeneration. It is mediated by dysregulation of extracellular matrix (ECM) and inflammation. In recent years, regenerative therapies have garnered attention for their potential to restore disc function by addressing the underlying biological alterations within the IVD. This review focuses on the comprehensive understanding of the anatomy and physiology of the IVD, highlighting its life cycle from embryonic development, and maturation to degenerative phenotype. We describe current treatments for managing LBP caused by IVD degeneration. This review emphasizes on the recent advancements in hydrogel engineering, highlighting natural, synthetic, and composite hydrogels and their application in ECM-targeted regenerative therapy for IVD degeneration. By exploring innovations in hydrogel technology, including improvements in crosslinking techniques and controlled degradation rates—we discuss how these materials could enhance IVD regeneration and potentially be used for the management of LBP. With their enhanced biomimicry, hydrogel-based ECM mimics offer a promising pathway for developing effective, durable therapies that address the root causes of disc degeneration, providing new hope for individuals living with chronic LBP.

## Introduction

Lower back pain (LBP) is a common condition that affects many people. Along with neck problems, it is the most prevalent spinal disorder, particularly in the aging population (Uysal et al., 2019). According to the World Health Organization, LBP is defined as pain between the lower edge of the ribs and the buttocks. It can be short-term (acute), medium-term (sub-acute), or long-term (chronic), and it can affect anyone (World health organization, 2023). It is the leading cause of activity limitation and absenteeism from work, creating a substantial medical and economic burden (Wu et al., 2020). In 1990, the prevalence of LBP affected approximately 377.5 million people, rising to 619 million by 2020. This number is expected to reach 843 million by 2050, largely due to population aging and growth (World health organization, 2023; Wu et al., 2020).

Degeneration of intervertebral discs (IVDs) is linked to around 40% of chronic back pain cases (Basatvat et al., 2023). IVD tissues are located between vertebral bodies, a crucial mechanical load-bearing structure that facilitates the functional articulation of the spine (Baumgartner et al., 2021). Nucleus pulposus (NP) tissues have a high water content, providing a crucial buffering function that helps maintain IVD height and spinal joint mobility. However, the water content of NP tissues in children is typically greater than 80%, but it decreases with age, dropping to as low as 70% in older individuals. This loss of water content is a major contributing factor to intervertebral disc degeneration (IVDD) (Salvatierra et al., 2011).

IVD degeneration is marked by a shift from anabolic to catabolic processes, leading to heightened extracellular matrix (ECM) degradation, IVD, and bone remodeling, as well as loss of hydration and altered spine biomechanics. Additionally, IVDD involves the release of proinflammatory cytokines from native disc cells, as well as the induction of angiogenesis and neoinnervation (Basatvat et al., 2023). Current treatments, including medication, rehabilitation, and spinal surgeries, aim to alleviate pain and are effective for short-term relief. However, they have limited long-term effectiveness in managing chronic symptoms and focus on symptom relief without restoring normal IVD function (Zhang et al., 2022; Schnitzer et al., 2004).

ECMs are complex, three-dimensional networks that play vital roles in tissue organization, remodeling, and regulation of cellular processes. These matrices are primarily composed of collagen, proteoglycans (which consist of glycosaminoglycans (GAGs), attached to a protein core), elastin, elastic fibers, laminins, fibronectin, and various other proteins and glycoproteins, including matricellular proteins (Karamanos et al., 2021). Despite making up only 1% of IVD volume, cells are essential for the synthesis and degradation of the ECM (Sheyn et al., 2019). Hydrogels are extensively utilized in biomedical engineering due to their favorable properties. Their mechanical characteristics closely resemble those of NP tissue, making them a focal point in recent research for treating IVDD. Hydrogels are three-dimensional hydrophilic polymers with high water content and excellent biocompatibility. Similar to the natural ECM, they serve as carriers for delivering drugs, proteins, and stem cells (Yan et al., 2021).

With IVDD being a major cause of lower back pain and disability, finding better treatments is crucial. ECM is essential for disc health, and new developments in hydrogel technology offer exciting possibilities for repair. By closely mimicking the natural ECM, hydrogel-based therapies could provide effective solutions for regenerating discs and tackling the underlying issues of degeneration. This review will examine the latest advances in hydrogel treatments, highlighting how they could improve disc health and offer hope for better management of back pain. Our goal is to explore these innovations and their potential to change the way we approach disc regeneration.

## Anatomy and physiology of the intervertebral disc

The vertebrate axial skeleton evolved to support the body and protect the spinal cord, comprising two main components: bony vertebrae, which develop from cartilage through endochondral ossification, and connective tissues, including IVDs, ligaments, and tendons. Acting as the spine’s shock absorber, the IVD plays a crucial role in maintaining spinal function by providing connectivity and cushioning between vertebral bodies (Williams et al., 2019). Made of fibrocartilage, each disc serves as the primary joint linking adjacent vertebrae in the spinal column and contains key components such as GAGs, collagen, and aggrecan. In the human spine, 25 IVDs are distributed across the cervical (7 discs), thoracic (12 discs), lumbar (5 discs), and sacral (1 disc) regions (Romaniyanto et al., 2022). Structurally, each disc consists of three main parts: the NP, a gel-like core that contains a high proportion of proteoglycans relative to collagen, specifically type II collagen; the annulus fibrosus (AF), a tough outer layer primarily composed of type I collagen with a higher collagen-to-proteoglycan ratio; and two cartilaginous endplates (CEP), which allow nutrient and fluid exchange between the disc and adjacent vertebrae (Uysal et al., 2019; Steinmetz and Benzel, 2016).

### Annulus fibrosus

The AF plays a crucial role in the biomechanical function of the intervertebral disc by encasing the NP, maintaining its containment, and ensuring proper intradiscal pressure during loading (Li et al., 2014). It maintains the NP’s hydrostatic pressure, controls the range of motion, and ensures the integrity of the motion segment (Stein et al., 2021). Unlike the NP and CEP, the AF is an inherently heterogeneous tissue, consisting of a series of concentric layers that encircle the NP (Li et al., 2014). The AF contains 65%–70% water, and its dry weight is composed of approximately 20% of proteoglycans, which play a key role in retaining water within the tissue, 50%–70% collagen, and 2% elastin (Isa et al., 2023; Newell et al., 2017).

The AF is divided into outer and inner regions, each with distinct cellular compositions. The inner AF contains rounded, chondrocyte-like cells and is primarily composed of type II collagen, which forms a fine meshwork that binds with proteoglycans, and aggrecan, which retains water (Steinmetz and Benzel, 2016; Li et al., 2014; Newell et al., 2017). It also has a higher concentration of proteoglycans (30%) and its collagen fibers contain 50%–100% more water than those in the outer AF (Steinmetz and Benzel, 2016). In contrast, the outer AF is densely packed with spindle-shaped fibroblastic cells and consists of approximately 70% type I collagen by dry weight, which enhances the tissue’s tensile properties and strength, making it stiffer and less flexible (Steinmetz and Benzel, 2016; Li et al., 2014; Newell et al., 2017). The outer AF has a lower concentration of proteoglycans (10%) and less water content in its collagen fibers compared to the inner AF.

Functionally, the inner AF acts as a transition between the dense, fibrous outer region and the gelatinous NP, while the outer AF anchors the vertebrae and stabilizes the NP, preventing herniation. The unique cellular and compositional traits of the inner and outer AF regions are vital to their distinct biomechanical functions (Steinmetz and Benzel, 2016). The AF becomes increasingly less vascularized toward its center, with blood vessels restricted to the outer third. Moreover, only the outer third receives sensory innervation, limiting pain perception and the ability to repair in the central regions (Moore and Dalley, 2018; Fournier et al., 2020).

### Nucleus pulposus

The NP is the soft, gel-like core of the intervertebral disc, rich in proteoglycans that aid in water retention and inhibit endothelial cell migration (Fournier et al., 2020; Bertolo et al., 2012). It constitutes approximately 30%–50% of the disc’s cross-sectional area (Steinmetz and Benzel, 2016). Although it has a relatively minor role compared to the AF in managing internal pressure and transferring load within the disc (Growney et al., 2017), the NP plays a crucial role in spinal flexibility and impact absorption. Its unique viscoelastic properties enable it to deform under compression and tension, efficiently managing forces and ensuring smooth spinal movement (Steinmetz and Benzel, 2016; Moore and Dalley, 2018; Xu et al., 2021).

The NP is highly hydrated, containing 70%–90% water, which enables it to generate hydrostatic pressure under compressive forces (Isa et al., 2023). Its dry weight is composed primarily of proteoglycans, which account for 35%–65% and play a key role in retaining water. Fine type II collagen fibrils, making up 5%–20% of the dry weight, form a supportive network within the NP, with sulfated GAGs embedded in this loose collagen structure (Newell et al., 2017; Borrelli and Buckley, 2020). The remainder of the NP consists of non-collagenous proteins and elastin (Newell et al., 2017). The cellular composition of the NP is heterogeneous, consisting of notochordal NP cells (NCs) and chondrocyte-like NP cells (CLCs). As the body ages, NCs gradually decline in number as and differentiate into CLCs. By around 10 years of age in humans, NCs are typically absent, leaving CLCs as the predominant cell type within the NP. This cellular shift contributes to the functional changes observed in the NP over time (Hagizawa et al., 2023).

Being avascular, the NP receives its nutrients via diffusion from blood vessels at the edges of the AF and the vertebral body (Moore and Dalley, 2018; Chen et al., 2017). The surrounding ligaments and CEP, connected to the spinal artery, are key sources of nutrient supply. NP cells produce proteins like Fas ligand, along with proteoglycans and sulfated GAGs (such as chondroitin sulfate), which prevent blood vessels from penetrating the NP by inducing the death of endothelial cells (Fournier et al., 2020; Chen et al., 2017). Additionally, NCs secrete anti-angiogenic factors such as Noggin and Chordin, which inhibit the formation of new blood vessels by blocking vascular endothelial growth factor signaling (Cornejo et al., 2015). These mechanisms ensure that the NP remains avascular, preserving its critical role in spinal health and function.

### Cartilaginous endplates

The IVD, the largest avascular structure in the body, relies entirely on diffusion for nutrient supply. A critical component in this process is the CEPs, thin layers of hyaline cartilage approximately 600 μm thick. These endplates are predominantly composed of type II collagen, GAGs, and water. Notably, the concentrations of GAGs and water are higher near the NP compared to the AF, enhancing the diffusion of essential nutrients. The unique composition of the CEPs enables them to act as both mechanical barriers, containing the pressurized NP, and metabolic facilitators, supporting the disc’s nutritional demands despite the absence of direct blood supply (Moon et al., 2013; Wang L. et al., 2021).

### Mechanobiology of the IVD

The IVD is a mechanically active structure that experiences compression, tension, shear, and fluid pressures during daily movements. Dynamic loading is essential for disc health, as it exposes IVD cells to a variety of mechanical cues that help maintain disc integrity, guide cellular behavior, and support spinal flexibility (Setton and Chen, 2006; Korecki et al., 2008; Neidlinger-Wilke et al., 2005). Mechanical forces regulate matrix synthesis and turnover by altering the biochemical environment through tissue compaction and fluid movement (Neidlinger-Wilke et al., 2005). Axial compression during daily activities leads to fluid outflow, which alters local ion concentrations and osmotic pressure. While moderate dynamic loading cycles (0.2–1 Hz) enhance nutrient diffusion and support matrix homeostasis, excessive loading can deplete proteoglycans, thereby reducing the osmotic gradient and compromising tissue function (Chan et al., 2011).

Experimental studies have shown region-specific responses to mechanical stimuli: cyclic strain enhances collagen II and aggrecan expression in AF cells, whereas intermittent hydrostatic pressure promotes collagen I and aggrecan expression in NP cells. Both loading types also suppress matrix-degrading enzymes such as MMP-2 and MMP-3, supporting matrix preservation and reducing catabolic activity (Neidlinger-Wilke et al., 2005). IVD cells, particularly in the NP, rely on glycolysis due to the disc’s low oxygen environment. However, limited glucose availability or impaired lactate clearance can lead to acidification and reduced cell viability (Fearing et al., 2018). Mechanical loading further influences both matrix turnover and cellular metabolism through pressure, fluid flow, and electrokinetic forces. Notably, NP cells respond optimally to low-to-moderate static compression, while excessive mechanical loading may induce catabolic activity and tissue degeneration (Chan et al., 2011; Fearing et al., 2018).

## From formation to degeneration: the lifecycle of intervertebral disc

### Embryonic development of the intervertebral disc

**FIGURE 1:**
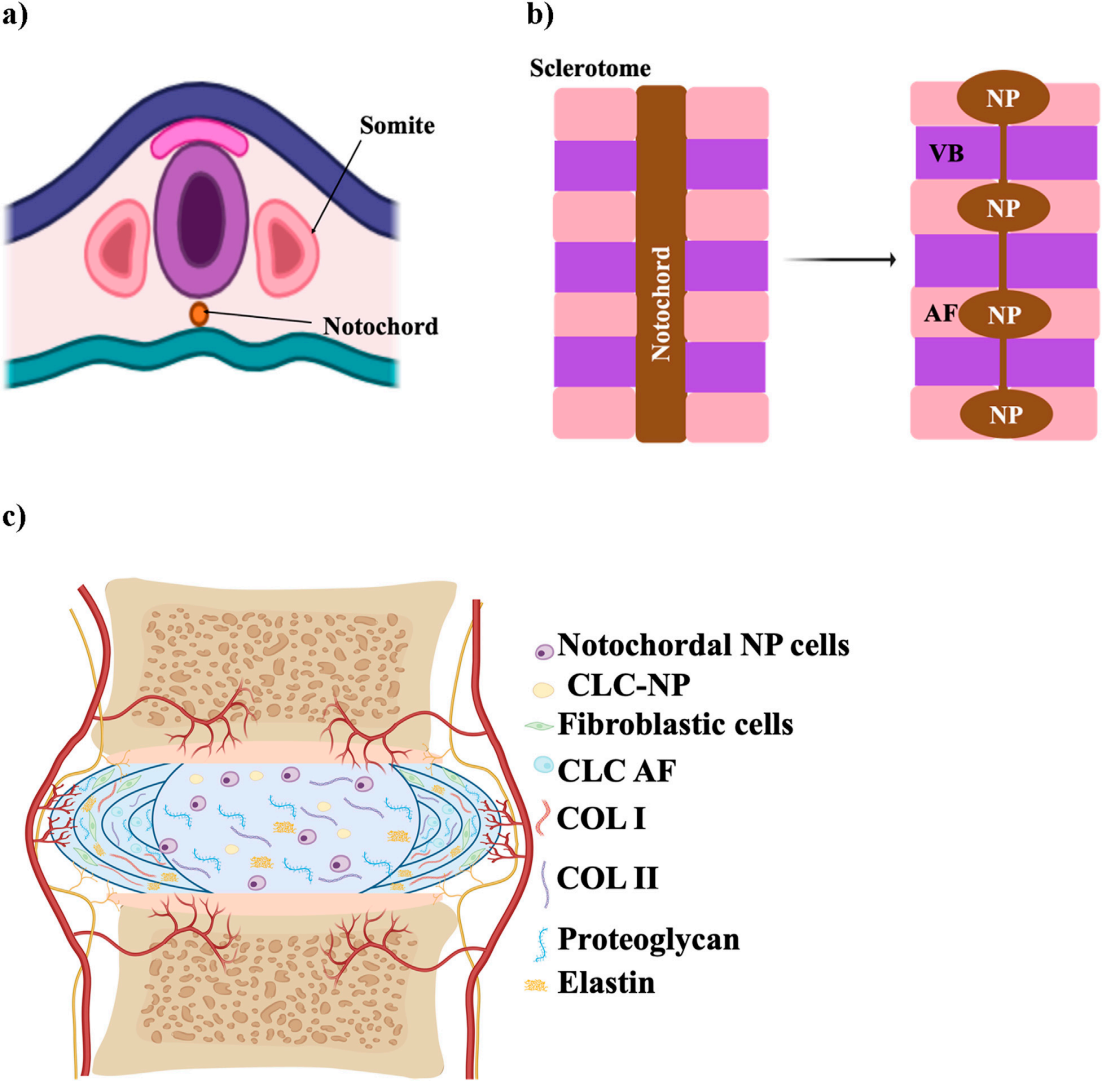
Schematic illustration of IVD development. **(a)** Early embryonic stage showing the notochord and sclerotome derived from somites. **(b)** Segmentation of the notochord, which will contribute to the formation of the NP within each IVD, while surrounding sclerotomal cells form the AF and vertebral bodies (VB). **(c)** Mature structure of the IVD, with a central NP surrounded by AF, CEP, and adjacent vertebrae. The schematic was created with BioRender.com.

During embryonic development, the IVD forms from the mesoderm (Hickman et al., 2022). The NP originates from the axial mesoderm-derived notochord, with its formation regulated by signals like T-brachyury, FoxA2, and Sonic hedgehog (Shh) (Hickman et al., 2022; Lawson and Harfe, 2017) as shown in Figure 1a, which show the early embryonic stage. In contrast, the AF, along with vertebrae, ligaments, and tendons, arises from the sclerotome of somites, which are transient structures contributing to the segmented vertebral column (Williams et al., 2019; Lawson and Harfe, 2017). Somites are transient structures that differentiate into the sclerotome, which forms the majority of connective tissues in the axial skeleton, including vertebrae, ligaments, and tendons (Williams et al., 2019) (Figure 1b). AF development is directed by specific signaling pathways, including TGF-β1 for matrix synthesis, Pax1 and Pax9 for sclerotome formation, and Wnt/β-catenin for AF differentiation (Hickman et al., 2022).

### Maturation and degeneration of the IVD

The mature structure of the IVD, consisting of AF, NP, and CEP is illustrated in Figure 1c. Degeneration of the IVD arises from a range of factors, including nutritional deficiencies, mechanical stress, injury or trauma, and genetic predisposition (Anjankar et al., 2015). It is associated with an imbalance in ECM homeostasis, marked by reduced ECM production and increased ECM breakdown (Isa et al., 2023), which can begin as early as the first decade of life when NCs shift to form CLCs, initiating degeneration in the NP (Isa et al., 2023; Swahn et al., 2024). In a healthy, youthful disc, NCs play a crucial role by secreting soluble factors that induce mesenchymal stem cells to differentiate into NP-like cells. These differentiated cells produce high levels of proteoglycans, resist collagen fiber expression, and avoid hypertrophy (Purmessur et al., 2011). However, as the disc matures, NCs diminish, and the NP loses its gelatinous structure, marking one of the first observable changes in the disc (Palacio-Mancheno et al., 2018).

**FIGURE 2:**
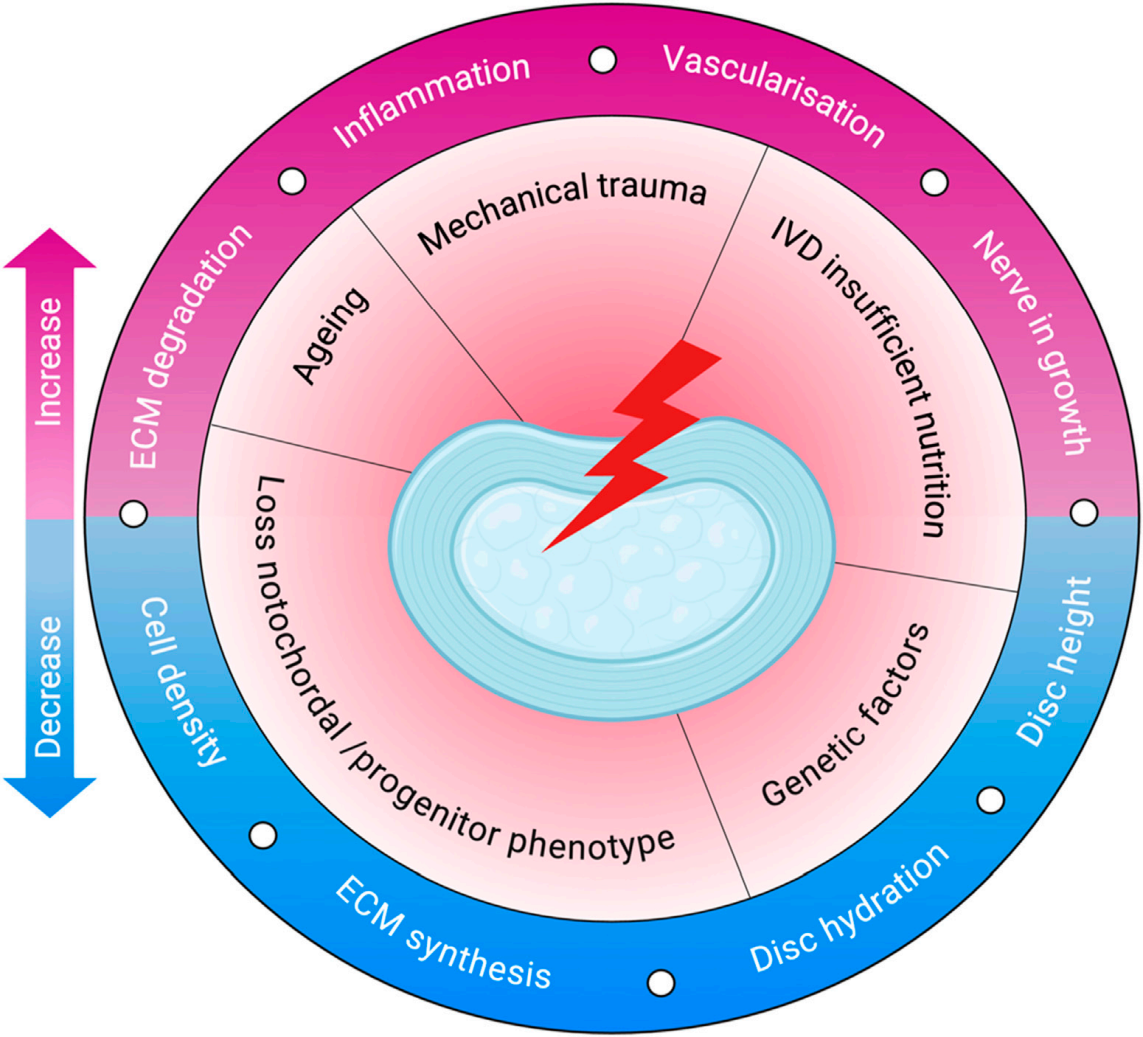
Pathophysiology of IVDD. The schematic illustrates key initiating factors—such as ageing, mechanical trauma, and poor nutrition—that drive degenerative changes in the disc. These lead to reduced cell density, ECM synthesis, and hydration, along with increased inflammation, ECM degradation, vascularisation, and nerve ingrowth. Created with BioRender.com.

The degeneration process involves biomechanical changes as well as alterations in the ECM and shifts in cellular activity as illustrated in Figure 2 (Rivera et al., 2022). In children, the NP is rich in water—over 80%—but this decreases with age, dropping to around 70%, contributing to disc dehydration and degeneration (Salvatierra et al., 2011; Yaltirik et al., 2019). As IVDD advances, the number of cells in the NP decreases, and their function becomes impaired, leading to an imbalance between ECM synthesis and degradation (Sheyn et al., 2019). Painful IVDD is driven by inflammation that alters glycosylation, leading to hyperinnervation and sensory sensitization, which ultimately results in discogenic pain (Mohd Isa et al., 2018). One of the key changes observed during this process is a shift from collagen II to collagen I within the disc (Sheyn et al., 2019).

Both genetic predispositions and environmental factors contribute to IVDD (Trefilova et al., 2021). Genetically, variations in genes such as Aggrecan can impact the IVD structure. Polymorphisms in genes related to catabolic processes, including matrix metallopeptidase 1, matrix metallopeptidase 2, matrix metallopeptidase 3, parkin RBR E3 ubiquitin-protein ligase, and proteasome 20S subunit beta 9, along with genes encoding Tissue Inhibitors of Metalloproteinases, can disrupt the balance between anabolic and catabolic activities, leading to disc degeneration (Dowdell et al., 2017). Environmental factors, such as lifestyle and chronic diseases, are also strongly linked to increased disc degeneration risk. Metabolic conditions like diabetes and hyperlipidemia are particularly noteworthy; in these cases, the degenerating IVD often exhibits an overactive tissue renin-angiotensin system. This overactivity accelerates NP cell aging, leading to heightened apoptosis, oxidative stress, and inflammation (Li et al., 2022).

In a healthy disc, nerve fibers are confined to the outer lamellae of the AF (Fournier et al., 2020). However, when disc damage occurs, growth factor production increases to support tissue repair, new blood vessels form, and granulation tissue develops. This damage also triggers neo-innervation, where new nerve fibers invade regions of the disc where they are typically absent. This nerve ingrowth, combined with the release of inflammatory mediators, contributes to the pain often associated with disc degeneration (Sakai and Grad, 2015). Degenerative NP cells fail to inhibit nerve growth and may even stimulate it. During inflammation, NP cells release elevated levels of pro-inflammatory cytokines, including TNF-α, IL-1β, IL-6, and IL-17 (Zàaba et al., 2025). These cytokines promote matrix degradation, trigger immune responses, and lead to increased nerve fibers and inflammatory mediators, exacerbating pain associated with disc degeneration (Sakai and Grad, 2015; Yang et al., 2024).

## Experimental models for intervertebral disc research

IVD treatment requires rigorous preclinical evaluation using *in vitro, ex vivo*, and *in vivo* models (Lazaro-Pacheco et al., 2023) summarized in Table 1. These models provide essential insights into the material’s biological performance and safety, ensuring a comprehensive understanding of degeneration mechanisms and therapeutic efficacy before advancing to clinical applications.

**TABLE 1:** Comparison of models used for studying intervertebral disc degeneration.

Model type	Species used	Degeneration induction methods	Advantages	Limitations	References
*In vitro*	Human/Animal disc cells (2D and 3D cultures)	Mechanical stress, Enzymes, Cytokines (biochemical), Hybrid (mechanical + biochemical)	Cost-effective, high-throughput, controlled conditions, ethical	Does not replicate IVD’s full complexity (e.g., avascularity, hypoxia, ECM structure)	(Rivera et al., 2022; Lazaro-Pacheco et al., 2023; Cao et al., 2021; Marinkovic et al., 2016; Vinken and Blaauboer, 2017; An and Masuda, 2006)
*Ex vivo*	Mouse, Rat, Rabbit, Bovine, Ovine, Caprine, Porcine, Human (cadaveric)	Proinflammatory cytokines, Mechanical injury, Degenerative loading, Enzymes	Preserves native disc structure and environment, reduces animal use, physiologically relevant	No systemic circulation, difficult to mimic low-nutrient conditions	(Salzer et al., 2023; Tang et al., 2022; McDonnell and Buckley, 2021b)
*In vivo*	Rodents, Rabbits, Dogs, Sheep, Pigs, Goats, Non-human primates	Surgical injury, Needle puncture, Enzymes, Mechanical loading	Replicates systemic interactions (vascular, immune), long-term assessment, clinically relevant biomechanics	High cost, ethical concerns, interspecies differences	(Tang et al., 2022; Poletto et al., 2022; Goel et al., 2020; Ribitsch et al., 2020; Kiani et al., 2022)

*In vitro* disc degeneration models—including mechanical, biochemical, and hybrid systems—offer controlled, cost-effective, and ethically favorable platforms for studying IVD biology. Mechanical models apply physical stress or disruption to simulate degeneration, while biochemical models use enzymes to mimic ECM breakdown; hybrid models combine both approaches to better simulate the multifactorial nature of degeneration (Rivera et al., 2022; Lazaro-Pacheco et al., 2023). Although 2D and 3D culture systems have been used to examine cellular responses under these conditions, they only partially replicate the native disc’s complex mechanical environment. These models provide a valuable setting to evaluate treatment properties, mechanisms of action, and therapeutic potential before clinical application (Cao et al., 2021). Furthermore, they enable the study of cytotoxicity, cell viability, and cellular behavior through various assays, such as MTT and Live/Dead staining (Marinkovic et al., 2016; Vinken and Blaauboer, 2017). Although these models are simpler than native disc structures, they have proven instrumental in identifying therapeutic targets and advancing the understanding of cellular behavior in disc degeneration. However, their inability to fully replicate the IVD’s avascular, hypoxic, and nutrient-deprived microenvironment remains a significant limitation (An and Masuda, 2006).

Degeneration in *ex vivo* models is typically induced by proinflammatory cytokines, mechanical injury, degenerative loading, enzymatic degradation, and less common techniques. Degenerative loading is the only method applied across all species, while cytokine-based induction is primarily used in small animal models (McDonnell and Buckley, 2021a). One key advantage of *ex vivo* models is their ability to preserve the native structure and cellular environment of the disc, providing more physiologically relevant conditions than standard *in vitro* cultures (Salzer et al., 2023). Discs from various species—such as mouse, rat, rabbit, bovine, ovine, caprine, porcine, and human—have been used in *ex vivo* models. Among these, bovine caudal (tail) discs are the most commonly utilized due to their availability, size, and structural similarity to the human disc, making them a widely accepted model in the field (McDonnell and Buckley, 2021a; Salzer et al., 2023). Degeneration in *ex vivo* models is typically induced through five main methods: proinflammatory cytokines, mechanical injury, degenerative loading, enzymatic degradation, and other less common techniques. Of these, degenerative loading is the only method applied across all species, while cytokine-based induction is predominantly used in small animal models (McDonnell and Buckley, 2021a). However, *ex vivo* models have limitations, such as difficulty mimicking the low-nutrient environment of human IVDs and lacking a circulatory system, which restricts vascular studies to *in vivo* models (Salzer et al., 2023; Tang et al., 2022).

In the absence of naturally occurring, reproducible IVDD in humans, various preclinical *in vivo* animal models have been developed to investigate the underlying mechanisms of disc degeneration and to evaluate potential therapies (Poletto et al., 2022). These models help simulate spinal biomechanics and degeneration processes that are relevant to human IVDD. Commonly used species include rodents, rabbits, dogs, sheep, pigs, goats, and non-human primates (Poletto et al., 2022; Goel et al., 2020). Rodents are the most widely employed due to their availability and ease of handling, with disc degeneration typically induced through surgical procedures and evaluated using histological methods. Study durations vary widely, from as short as 1 week in small animals to more than 104 weeks in larger species. Nevertheless, 4 and 12 weeks are the most frequently used time points across studies (Poletto et al., 2022). Although *in vivo* animal studies have advanced biomedical research, they are associated with ethical concerns and high costs (Ribitsch et al., 2020; KIANI et al., 2022).

## Current treatments

IVD disease can be managed through two primary approaches: conservative and surgical methods. Conservative treatment, which includes pharmacological interventions, physical therapy, and patient education, is typically the first-line approach. If these measures fail or the condition worsens, surgical intervention may be required (Isa et al., 2023; Ishiguro et al., 2019).

Conservative management involves both non-pharmacological strategies, such as physical therapy, self-management, and complementary medicine, and pharmacological options. Pharmacological treatment is recommended if non-pharmacological methods are ineffective (Isa et al., 2023; Mohd Isa et al., 2023; Foster et al., 2018; Zheng et al., 2023). Oral non-steroidal anti-inflammatory drugs (NSAIDs) are commonly used for managing uncomplicated lower back pain, providing short-term relief by inhibiting cyclooxygenases (COX-1 and COX-2) and reducing inflammation (Foster et al., 2018; Vaudreuil et al., 2017). However, they do not address the underlying degeneration and often become less effective as IDD progresses, making them a palliative rather than curative treatment (Isa et al., 2023; Foster et al., 2018).

Surgical intervention is typically considered the last resort for managing degenerative disc disease (Ishiguro et al., 2019). Options include simple decompression surgery, fusion surgery, IVD replacement, and endoscopic resection of diseased IVD tissue (Zhang et al., 2022). While effective in pain relief, these procedures are invasive, carry risks of complications, and tend to be more costly than non-surgical treatments (Ishiguro et al., 2019). UK guidelines recommend disc replacement and spinal fusion surgery for LBP only within randomized trials, limiting fusion surgery to these studies (Foster et al., 2018). Additionally, while fusion surgery, which eliminates motion between spinal segments, can relieve pain, it may also increase the risk of degeneration in adjacent spinal segments (Rizvi, 2015).

## Biomaterial approach for intervertebral disc repair: rationale of hydrogel-based extracellular matrix restoration

Innovative treatments are rapidly advancing, focusing on disrupting the biological pathways involved in IVDD (Rizvi, 2015). According to the American National Institute of Health, a biomaterial is any substance, whether synthetic or natural, used to augment or replace tissues, organs, or bodily functions either partially or fully to improve or sustain an individual’s quality of life (Bakshi et al., 2020). Both *in vitro* and *in vivo* studies have highlighted exciting developments in regenerative medicine that utilize biomaterial technologies for IVD repair (Rizvi, 2015).

Biomaterials can be designed as single or combination therapies, often functionalized with cells, therapeutic agents, or other substrates to enhance their effectiveness (Isa et al., 2018). Notably, biocompatible ECM-based biomacromolecules offer distinct advantages due to their non-cytotoxic nature and their ability to provide crucial instructive cues through the regulation of cellular signaling (Mohd Isa et al., 2022). This supports effective tissue development, maintenance, and regeneration, thereby enhancing the potential of these biomaterials for IVD repair and the treatment of lower back pain (Mohd Isa et al., 2023).

For effective tissue regeneration, biomaterials must be designed to support cell attachment, migration, growth, and differentiation, while also preventing chronic inflammatory responses or rejection by surrounding tissues. Scaffolds are three-dimensional constructs that mimic the native ECM, play a vital role in this process. Typically made from biodegradable and biocompatible polymers, these scaffolds must degrade at a rate that aligns with the tissue’s healing or growth rate. Hydrogels represent a specialized type of scaffold (Echeverria Molina et al., 2021). Hydrogel-based scaffolds are a crucial class of scaffolds because their mechanical properties can be customized to closely resemble those of natural tissues (El-Sherbiny and Yacoub, 2013). Table 2 summarizes the type of biomaterials and their key findings in the *in vitro* and *in vivo* models. We have also updated the clinical trials on the use of biomaterials with or without stem cells or drugs for IVD repair targeting LBP in degenerative disc disease patients (Table 3).

**TABLE 2:** Various biomaterials used in IVD regeneration and their key findings.

Biomaterial	Crosslinker	Composition	Model	Mechanism of action	Analysis and result	Ref
HA with type II collagen	4S-Star PEG	Weight ratio HA to collagen II: 1:9 and 4.5:9	*In vitro*	Mimics the NP microenvironment, supporting human Wharton’s Jelly -MSC viability and differentiation through its biocompatible, stable, and degradable 3D matrix	Significantly higher swelling capacity in HA/collagen II 4.5:9 compared to HA/COLII 1:9. Both formulations reached stability in an aqueous solution from day 21 up to 1 month of incubation at 37°C. Degradation analysis with type II collagenase demonstrated a time-dependent increase in the degradation percentage for both formulations	Mohd Isa et al. (2023)
		Weight ratio HA to collagen II 1:9 Induction TGF-β3	*In Vitro*		Human Wharton jelly-derived mesenchymal stem cells in hydrogel differentiated into NP-like cells with increased SOX-9, while 2D culture led to fibroblastic-like cells. Viability improved over time, indicating hydrogel biocompatibility	
Collagen cryogel	1-ethyl-3-(3-dimethylaminopropyl-carbodiimide hydrochloride/N-hydroxy-succinimide	Acidic Collagen (4wt%, pH 4.0) Induction TGF-β3	*In vitro*	Restores disc structure, retains water, and promotes regeneration, relieving pain and maintaining IVD integrity	Alginate shape memory and collagen cryogel demonstrated similar physical properties in terms of water absorption, compressive properties and shape memorability	Koo et al. (2023)
Alginate shape memory structure	CaCl_2_	Sodium Alginate (4 wt%) Induction TGF-β3		Absorbs water, changes shape in response to temperature or pH, and maintains mechanical properties. It promotes cell migration, proliferation, and matrix restoration		
			*In Vitro*		Cells remained viable in both hydrogels, with higher activity in CG than A-SMS. CG also induced more efficient and uniform chondrogenic activity	
		Scaffold Biomaterial with Hyaluronic Acid Scaffold Biomaterial Volume: 8ul HA Composition 1 w/v% in PBS, 15 μL	*In Vivo*		The CG group exhibited a higher withdrawal threshold, indicating reduced mechanical allodynia. MRI T2-weighted images showed better disc hydration in CG. Histology revealed greater NP area, cell number, and preserved disc structure. CG had lower histological grading scores, higher type II collagen and aggrecan, and lower type I collagen, suggesting enhanced extracellular matrix regulation. It also showed increased transcription factors Brachyury and Tie-2, indicating more NP cells. Additionally, CG downregulated proinflammatory cytokines, neurogenic factors, and catabolic enzymes, potentially reducing discogenic pain and preserving ECM.	
Decellularized nucleus pulposus matrix (DNPM) and chitosan hybrid hydrogel	physical crosslinking	2.5% DNPM, 1.5% Chitosan in 3% acetic acid	*In vitro*	DNPM promotes nucleus pulposus regeneration by providing a biomimetic environment that supports cell adhesion, migration, and differentiation. The DNPM mimics the natural extracellular matrix, enhancing cell interaction, while the chitosan hydrogel serves as a biocompatible scaffold that facilitates sustained release of growth factors	SEM Analysis: Smooth, porous structure, good connectivity FITR: collagen and polysaccharides Compression: elastic, fails at ∼70% strain Rheology: Stable storage modulus, good elasticity. pH: Neutral (7.1–7.3), supports cell growth.	Ma et al. (2024)
		DNPM/chitosan hydrogel mixed with GDF5-loaded PLGA microspheres	*In vitro*	PLGA microspheres provide a controlled, sustained release of GDF5, which promotes chondrogenic differentiation of NP stem cells and supports the regeneration of NP.	SEM: uniform spherical GDF5 microspheres (50–160 μm, avg. 110 μm) Encapsulation efficiency: 75.1% Release: slow release, plateau at day 10 Degradation: 20% residual mass after 24 days	
			*In vitro*		The composite hydrogel with GDF5-loaded microspheres enhanced chondrogenesis, with the nucleus pulposus stem cell showing the highest COL2A1 expression and secretion at 21 days	
			*In vivo*		GDF5/CH + NPSC hydrogel showed the best IDD repair, with the highest MRI signal, mildest degeneration, and highest COL2A1 expression	
Genipin-enhanced fibrin hydrogel combined with an engineered silk scaffold	Genipin	Fibrinogen, thrombin, genipin, DMSO. Fetal calf serum and *ε*-aminocaproic acid	*In vitro*	act as a crosslinked filler, fills the injury in the AF, while the silk scaffold provides additional support and structural integrity	Genipin combined with DMSO completely inhibited mitochondrial activity at all tested concentrations	Frauchiger et al. (2018)
			In *ex vivo*		No herniation in any loading condition, disc height not restored, matrix and DNA content similar to healthy control, and genipin safe in organ culture	
Mucin-derived gels	Tetrazine and norbornene click chemistry	Bovine Submaxillary Mucin, Tetrazine-amine, Norbornene-amine, EDC, NHS, MES buffer, and PBS.	*In vivo*	Immune modulation and protection against immune infiltration	Prevent fibrous encapsulation and macrophage infiltration in the mouse model. In the rat tail IVD degeneration model, Muc-gel injection prevents degeneration for up to 24 weeks post-operation. Mechanistically, Muc-gels attenuate immune cell infiltration into the NP, protecting against immune attack following microdiscectomy	Wang et al. (2024c)
chitosan/PEG hydrogel	Dual crosslink: Schiff base reaction and photo-crosslinking	chitosan, PEG, methacrylic anhydride, lithium phenyl-2,4,6-trimethylbenzoylphosphinate, and 1-(3-dimethylaminopropyl)-3-ethylcarbodiimide hydrochloride	*In vitro*	Rapid *in situ* seal at the defect site through photo-crosslinking and Schiff base reactions, providing mechanical support and physical plugging	Low cytotoxicity was observed when nucleus pulposus (NP) cells were cultured with the hydrogel	Huang et al. (2023)
			*In vivo*		Hydrogel sealed the IVD defect, reducing disc height loss and matrix degradation while preserving NP and AF structures in rat tail model	
Electrospun biodegradable poly (ε-caprolactone) membranes	N/A	The membrane was produced in three different fibres diameters (thin, medium, and thick), prepared by electrospinning Poly (ε-caprolactone) dissolved in solvents such as chloroform and methanol	*In vitro*	Provide mechanical support in tissue engineering by forming a structural scaffold	Membranes exhibited increased crystallinity and ester bond degradation over time. The modulus increased in the first loading cycle, then varied with subsequent cycles based on strain and membrane type. The elastic range improved with strain, and the modulus was within the lower range of human annulus fibrosus tissue, showing potential for sealing damaged annulus fibrosus	Alexeev et al. (2021)
PVA with a polyvinyl pyrrolidone	sodium trimetaphosphate	PVA: polyvinyl pyrrolidone ratios of 1:1 and 1:3 were used	*In vitro*	The thixotropic, injectable 3D network forms a stable structure that remains injectable due to chemical cross-linking with trisodium trimetaphosphate	The 1:1 Polyvinyl alcohol- polyvinyl pyrrolidone scaffold showed favourable viscoelasticity, no cytotoxicity, and supported chondrocyte adhesion and proliferation, making it a promising NP replacement	Leone et al. (2019)
PEG with decellularized notochordal cell-derived matrix	PEG-diurethane	8-arm-PEG-vinyl sulfone, PEG-diurethane-dithiol crosslinker, and decellularized notochordal cell-derived matrix	*In vitro*	leveraging the regenerative properties of the decellularized matrix along with the mechanical tunability of PEG hydrogels	Tunable stiffness, sustained release of decellularized notochordal cell-derived matrix, and high viability of bone marrow stromal cells, but notochordal cells lost activity over time	Schmitz et al. (2023)

**TABLE 3:** Updates on clinical trials used biomaterial to regenerate IVD.

Trial ID	Study design	Intervention	Group	Sample size	Country	Study period	Primary outcome	Remarks	Ref
NCT06778447	Prospective, multi-center, single-arm, open-label study	Via Disc NP	Experimental Group (VIA Disc NP – Intradiscal Injection)	60	USA	17 February 2025 - June 2027	effectiveness, assessed by the proportion of participants achieving at least a 30% reduction in back pain (Visual Analogue Scale) VAS score at 6 months, and safety, evaluated by the incidence of investigational-product-related adverse events (AEs, SAEs, UAEs) over 24 months	Status: recruiting participants Inclusion: aged 22–85 years and diagnosed with early to moderate degenerative disc disease (Modified Pfirrmann Grade 3–7). They should have experienced chronic axial low-back pain (with or without non-radicular leg pain) for at least 6 months, unresponsive to 3 months of conservative care With visual analogue scale (VAS) pain scores of 40–90 mm and Oswestry Disability Index (ODI) scores of 40–80. A positive hip flexion test and intolerance to prolonged sitting are required. Participants must provide informed consent and be able to comply with study procedures	Disc Genics (2023)
NCT03347708	Randomized, controlled, parallel-assignment, double-blind interventional trial	Sodium Hyaluronate	1. High-dose discogenic cells + sodium hyaluronate vehicle 2. Low dose discogenic Cells + sodium hyaluronate 3. Sodium Hyaluronate alone (Control) 4. Saline solution alone (Placebo control)	60	USA	26 February 2018, - 10 November 2022	Safety was assessed by the incidence of grade 2 or greater AEs and SAEs over 2 years, and efficacy was measured by pain reduction using the VAS over 1 year	Status: completed- no results posted Inclusion Participants must be aged 18–75 and have early to moderate IVDD with a Modified Pfirrmann Grade of 3–7. They must have experienced chronic low back pain for at least 6 months that has not responded to 3 months of conservative care. Additionally, they must have an LBP score of 40–90 mm on the VAS and an ODI score of 30–90 Exclusion: multiple symptomatic lumbar discs, radiculopathy, cauda equina syndrome, previous lumbar surgery, fractures, dynamic instability, grade 2+ spondylolisthesis, Type III Modic changes, full-thickness annular tears, facet pain, communicable diseases, significant systemic conditions, and those deemed unsuitable by the investigator	DiscGenics and Inc (2023)
NCT06011551	Randomized, controlled, parallel-assignment, single-blind interventional trial	ReGelTec HYDRAFIL	Experimental: Treatment Sham Comparator: Control	225	USA	January 2025 to November 2028	Composite Endpoint of Clinical Success: meeting all five study outcomes (function, SAEs, SSIs, intercurrent events, and radiographic findings) at 12 months	Status: Recruiting Inclusion: male or female aged 22 to 85, with chronic LBP, due to IVDD or at least 6 months. Participants must have one or two symptomatic discs with Grade 4 to 8 degeneration on MRI and have not experienced relief from conservative care for 6 months. Participants must be able to fully comply with the study protocol Exclusion: history of infections, prior back surgery, spinal instability or stenosis, major psychiatric disorders, substance abuse, severe osteoporosis, or cancer. Pregnant women, individuals with imaging contraindications, or any comorbid condition affecting safety or outcomes are also excluded	ReGelTec and Inc (2025b)
NCT04727385	Adaptive-design, interventional, open-label, single-arm trial with sequential cohort initiation based on DSMB safety review	Double cross-link microgel	One disc level cohort: 5 patients with only one disc to be treated Two-disc level cohort: 5 patients with two discs to be treated One- or two-disc level cohort:10 patients with either one or two discs to be treated	20	France	September 2020- October 2021	Evaluate safety and effects by tracking adverse events, serious adverse events, neurological changes, and MRI-based assessments of nucleus water content, intervertebral height, DXM gel position, and adjacent tissue modifications over 24 weeks	Status: Unknown Inclusion: male and female aged between 18 and 55, with chronic discogenic low back pain (ODI 30%–60%), MRI-confirmed Pfirrmann grade II/III disc degeneration (L1-S1), and no severe depression. Must comply with study procedures Exclusion: Nerve compression, severe disc degeneration (Pfirrmann IV/V), prior lumbar surgery, spine deformities, fractures, infections, BMI >35, psychiatric issues, substance abuse, pregnancy, or MRI contraindications	Gelmetix (2021)
NCT06325566	Prospective, multicenter, randomized, double-blind, controlled study with parallel assignment	Rexlemestrocel-L combined with hyaluronic acid	Experimental Group (Rexlemestrocel-L + HA): Participants receive a 2.0 mL injection containing approximately 6 million rexlemestrocel-L cells mixed in a 1:1 ratio with HA) solution as an intradiscal injection Control Group: Participants receive a saline solution injection adjacent to the index disc	300	USA	July 2024 - October 2027	Change in daily average LBP from baseline at 12 months, assessed using the VAS, and the incidence of adverse events and serious adverse events over 24 months	Status: Recruiting Inclusion: have moderate to severe LBP for at least 6 months, failure of at least 3 months of conservative care, and moderate radiographic degeneration of a single IVD from L1 to S1 suspected of causing chronic low back pain Exclusion: prior stem cell or biological treatments, LBP duration outside 6–60 months, lack of conservative treatment, use of immunosuppressants, osteoporosis, substance abuse, severe psychiatric conditions, neurological deficits, structural spinal surgeries, sacroiliac or facet joint pain, multiple painful levels, full-thickness annular tears, and mild or extreme LBP.	National Library of Madicine (2025)
NCT04530071	Randomized, double-blind, placebo-controlled, single-dose, phase 1/2a, multi-center study	Allogeneic umbilical cord-derived mesenchymal stem cells (CordSTEM-DD) combined with HA and saline.	Group 1: CordSTEM-DD (0.7 × 10^7 cells) combined with HA + saline Group 2: CordSTEM-DD (2.1 × 10^7 cells) combined with HA + saline Group 3: Placebo Comparator Group: HA + saline + placebo comparator	36	Korea	September 2020 - April 2023	Evaluation of treatment-emergent AEs during the study period. In Stage 1, AEs will be assessed over 28 days, while in Stage 2, AEs will be monitored for up to 12 months	Status: completed. The results have been submitted to ClinicalTrials.gov but are not yet publicly posted Inclusion: Adults aged 19–69 with chronic low back or hip pain for ≥6 months, unresponsive to conservative treatment, and MRI-confirmed lumbar disc degeneration. Must have a VAS ≥40 mm and ODI ≥30% Exclusion: BMI ≥30 kg/m^2^, severe spinal issues, prior spinal surgeries, ongoing high-dose opioid use, neuralgia, or autoimmune diseases. Excludes those on immunosuppressants, biological treatments, or PRP therapy, as well as pregnant/breastfeeding women or those with psychiatric issues	CHABiotech CO. Ltd (2023)

A hydrogel is a three-dimensional network of polymers that can absorb and hold significant volumes of water, saline, or biological fluids. These materials are inherently hydrophilic due to the presence of hydrophilic functional groups in their polymer chains, which attract and retain water (Zain et al., 2018). After absorbing water, the hydrogel swells and holds this expanded state even under pressure. Rather than releasing water as a free-flowing liquid, it gradually releases retained moisture as water vapor, allowing for controlled, slow diffusion through the gel (Zain et al., 2018; Rop et al., 2019). The stability of the hydrogel and its resistance to dissolution in water comes from the cross-links that interconnect the polymer chains. These cross-links form a robust network structure that keeps the gel intact and prevents it from dissolving (Zain et al., 2018). Hydrogels can be fabricated using either chemical or physical crosslinking methods (Zhang et al., 2020).

Hydrogels are increasingly being researched for treating IVD degeneration due to their ability to mimic the ECM and mechanical properties of NP tissue (Zheng and Du, 2021). An effective hydrogel for NP regeneration should be injectable, have a suitable gelation rate to prevent leakage, possess high mechanical strength and an appropriate degradation rate, provide swelling pressure under various loadings, support cell proliferation, and matrix deposition, and be biocompatible to minimize adverse effects after implantation (Gan et al., 2017). Hydrogels are designed to replicate the biophysical characteristics of the native IVD ECM, thereby enhancing their ability to support cellular function and regeneration (Wang et al., 2024a). The high water content of hydrogels facilitates ion and nutrient diffusion, closely resembling native disc conditions (Blache et al., 2022; Desai et al., 2024a). Ionically cross-linked systems can further regulate cell volume and promote cartilage-like ECM production, aiding tissue resilience under mechanical stress. These hydrogels offer tunable stiffness and viscoelasticity, which are critical cues for mechanotransduction and cellular behavior under dynamic physiological conditions (Wang et al., 2024a).

Stiffness plays a pivotal role in regulating cell responses such as spreading, proliferation, migration, and differentiation. By modifying the crosslinking density and polymer composition, hydrogel stiffness can be finely adjusted to mimic the evolving ECM during development or degeneration. This tunability enhances the regenerative potential of mesenchymal stem cells (MSCs), aligning their function with the microenvironment’s mechanical state (Gan et al., 2023). Viscoelasticity, another essential biophysical property, allows hydrogels to absorb compressive forces while maintaining structural integrity. This capacity supports long-term implantation and provides a mechanically supportive environment for cells subjected to physiological loading (Zhang et al., 2023a; Wang et al., 2024b). Furthermore, hydrogels that undergo cyclic strain or dynamic mechanical loading have been shown to improve nutrient transport through the generation of fluid pressure gradients. This loading enhances both convective and diffusive flow, leading to increased solute penetration and elevated wall-shear stress on embedded cells. A validated proelastic model confirmed that such mechanical stimuli improve nutrient delivery, supporting sustained cell viability and function within the scaffold (Vaughan et al., 2013).

## Classification of hydrogels: innovation and applications

**FIGURE 3:**
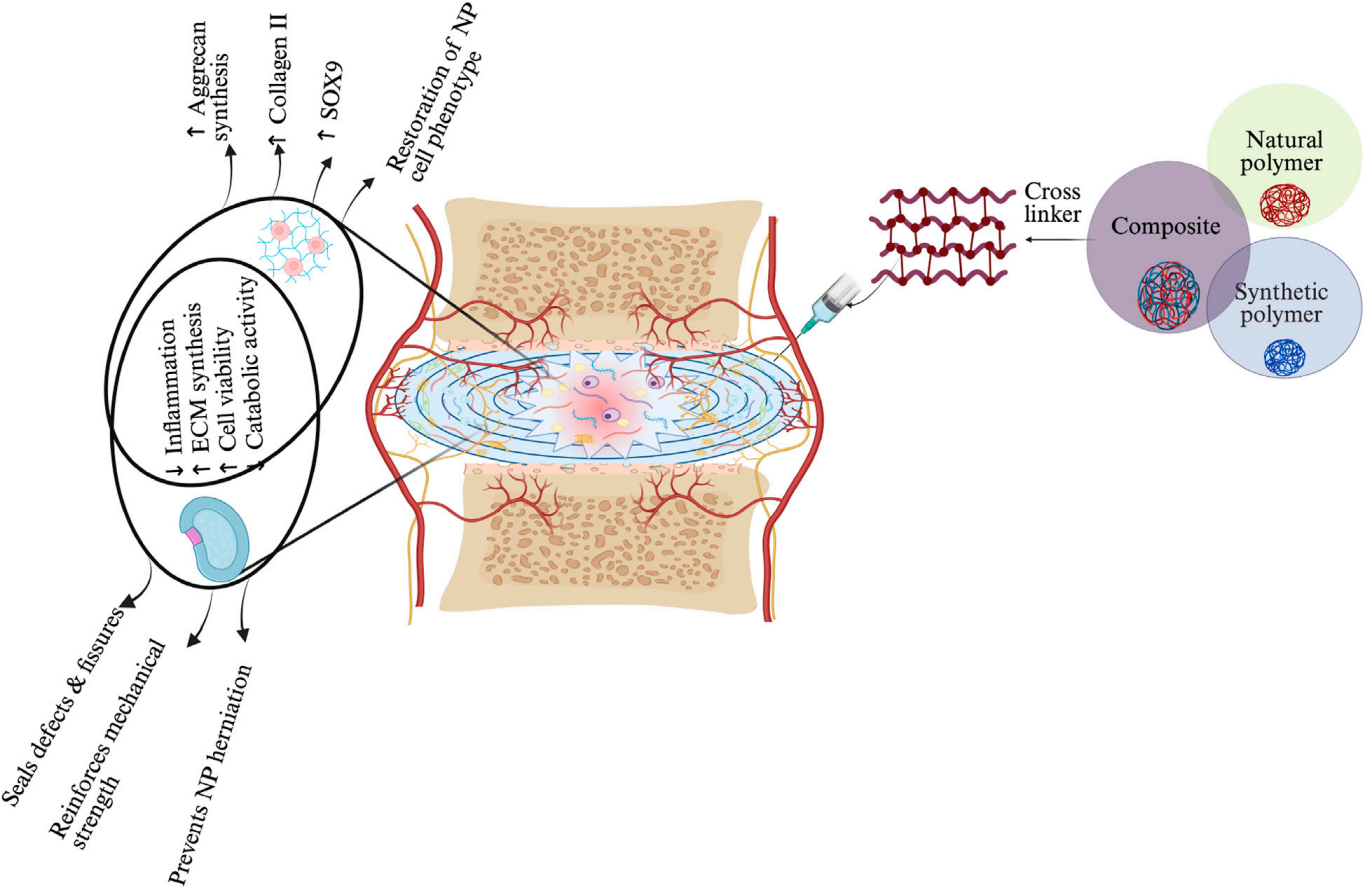
Schematic representation of hydrogel-based therapeutic strategies for IVD regeneration, highlighting material–cell interactions and mechanisms of action in NP and AF regions. Three main hydrogel categories—natural, synthetic, and composite—are illustrated, each offering distinct properties: natural hydrogels support biocompatibility, synthetic hydrogels enable tunable mechanics, and composites integrate both. Upon injection, the hydrogel modulates the IVD environment by reducing inflammation, promoting ECM synthesis, enhancing NP cell viability, and restoring NP-specific phenotype (↑SOX9, ↑Collagen II, ↑Aggrecan). In the AF region, the hydrogel reinforces mechanical strength, seals defects, and prevents NP herniation. These combined effects contribute to disc regeneration by restoring structure, hydration, and function. The schematic was created with BioRender.com.

Hydrogels can be classified into three categories based on their source: natural, synthetic, and composite hydrogels (Li et al., 2021) (Figure 3).

### Natural hydrogel

Natural hydrogels are derived from biopolymers such as polysaccharides and proteins, making them highly favorable for biomedical applications due to their biocompatibility, biodegradability, and bioactivity (Catoira et al., 2019; Varghese et al., 2020). Prominent examples of polysaccharides include hyaluronic acid (HA), alginate, chondroitin sulfate, chitosan (CHI), cellulose, and agarose, while key proteins include collagen, gelatin, and fibrin (Zhao et al., 2023). These natural polymers are especially valued in tissue engineering and regenerative medicine for their biodegradability, biocompatibility, and availability (Brinkmann et al., 2023).

Their natural composition promotes essential cellular functions and, due to their biocompatibility, low toxicity, and enzymatic degradability, they are widely used in regenerative medicine. These characteristics make natural gels particularly suitable for the repair and regeneration of the nucleus pulposus, as they can effectively support tissue healing and integration (Zheng and Du, 2021; Gan et al., 2017; Varghese et al., 2020). However, their weak mechanical properties limit their effectiveness in IVD regeneration when used alone, necessitating their combination with other materials for optimal performance (Wu et al., 2023).

#### Hyaluronic acid hydrogel

**FIGURE 4:**
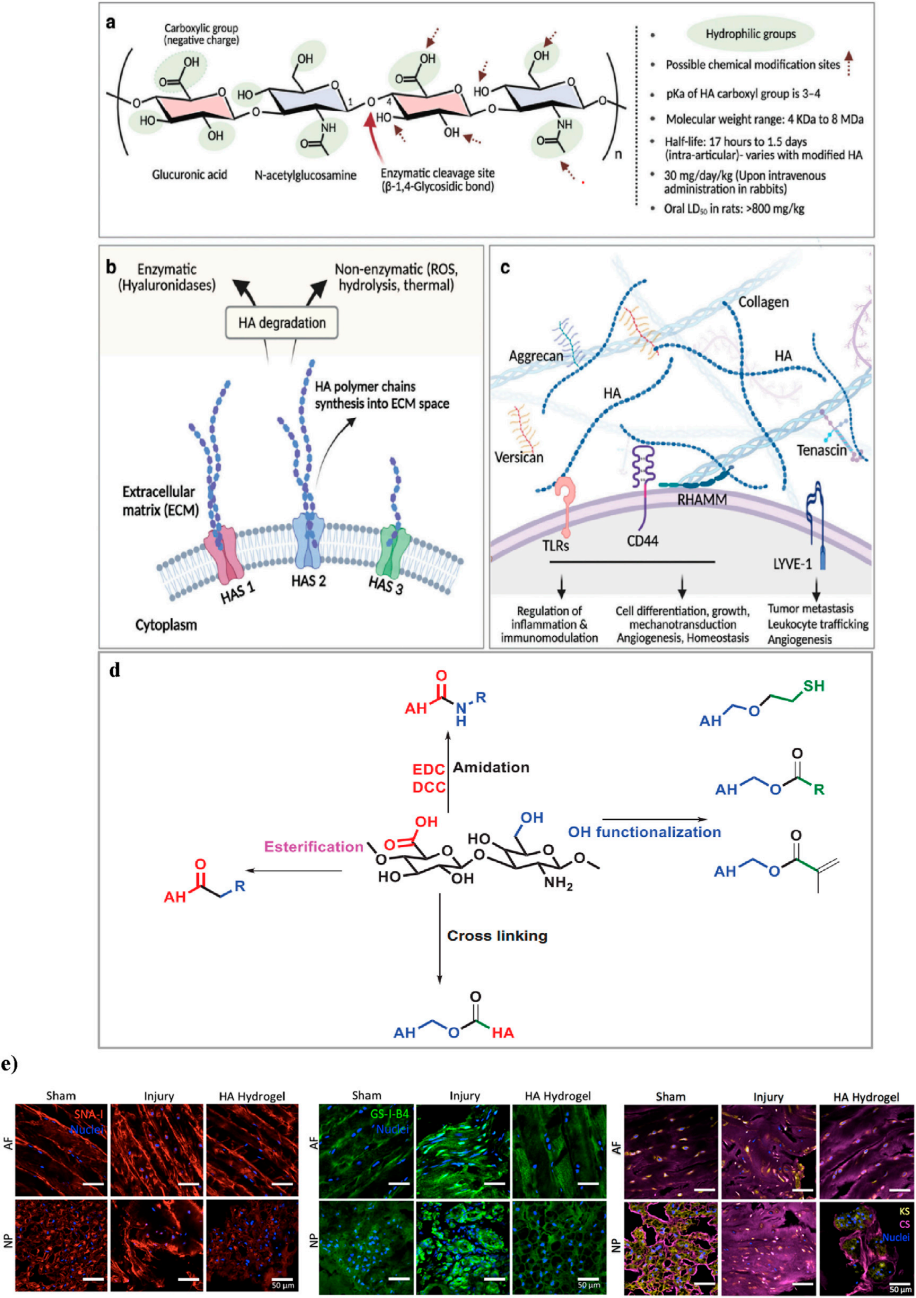
**(a)** Chemical structure of HA, highlighting its hydrophilic groups, enzymatic cleavage sites, and possible chemical modification points. **(b)** synthesis of HA by synthase enzymes (HAS1, HAS2, HAS3) and its degradation via enzymatic (hyaluronidases) and non-enzymatic (ROS, hydrolysis, thermal) pathways. **(c)** HA’s interactions in the ECM, forming networks with proteoglycans and binding to cell surface receptors. Figure adapted from Vaudreuil et al. (2017). **(d)** This schematic illustrates key chemical modifications of HA, including esterification, amidation, and hydroxyl functionalization. Esterification alters hydroxyl (-OH) groups. Amidation modifies carboxyl (-COOH) groups using carbodiimide chemistry (EDC/DCC). Hydroxyl functionalization introduces reactive groups such as thiols (-SH) and esters. Additionally, crosslinking strategies improve mechanical properties and stability, making these modifications essential for hydrogel engineering and tissue regeneration. Figure adapted from (Rizvi, 2015). **(e)** Effects of HA-hydrogel implantation on glycosylation in the injury-induced pain model. SNA-I (red label) and GS-I-B4 (green label) binding to α-(2,6)-linked sialic acid and α-galactose, respectively. Expressions of chondroitin sulfate (purple label) and keratan sulfate (yellow label) were denoted in the sham control, untreated injury and HA-hydrogel-treated injury groups, in AF and NP tissues. Figure adaptation from Mohd Isa et al (Mohd Isa et al., 2018).

Hyaluronic acid (HA) hydrogels provide ideal scaffolds for biomedical applications, supporting cell infiltration and nutrient diffusion essential for tissue regeneration (Gholamali et al., 2024). The native linear form of the long-chain HA biopolymer consists of repeating disaccharide units, where β-1,4-D-glucuronic acid and β-1,3-N-acetyl-D-glucosamine effects linked by β-glycosidic bonds (Figure 4a). Forming a gel-like matrix capable of retaining water and influencing tissue mechanics. Its molecular weight can be more than 4 MDa, which can affect its hydrodynamic radius and biomechanical properties, as the molecular weight of HA rises (from 7 to 700 kDa), its hydrodynamic radius expands from 3 to 54 nm. HA synthesis by enzymes (HAS1, HAS2, HAS3) and degraded by hyaluronidases or reactive oxygen species. It interacts with ECM components and binds to receptors like CD44, RHAMM, LYVE-1, and TLRs, regulating cell signaling, angiogenesis, immunity, and mechanotransduction (Kotla et al., 2023; Vieira et al., 2017) (Figures 4b,c).

HA is vital to many biological processes, its rapid degradation and poor mechanical stability limit its effectiveness in long-term therapeutic applications (Gholamali et al., 2024; Faivre et al., 2021). Cross-linking methods influence hydrogel strength, swelling, and degradation (Gholamali et al., 2024). HA, crosslinking can occur by directly adding a cross-cross or by pre-modifying HA chains with functional groups for crosslinking. The disaccharide units of HA have three modifiable sites: the carboxyl, hydroxyl, and N-acetyl groups. Carboxyl modification typically involves amide formation using coupling reagents like DMTMM or carbodiimide derivatives (e.g., EDC) with activators such as HOBt, NHS, or sulfo-NHS. Hydroxyl groups undergo oxidation (NaIO_4_), hemiacetal or ether formation (BDDE: 1,4-butanediol diglycidyl ether, DVS: divinyl sulfone), and esterification. N-acetyl groups can be modified via deacetylation and amidation (Pérez et al., 2021) (Figure 4d). In addition, HA-based hydrogels are crosslinked through physical, covalent, or dynamic covalent methods, enhancing polymerization efficiency and properties (Luo et al., 2023).

*In vivo*, HA hydrogel has demonstrated significant efficacy in alleviating pain associated with IVDD. In a rat tail model of disc injury, HA hydrogel significantly reduced nociceptive behavior, correlating with the downregulation of nociception markers and inhibition of hyperinnervation. Additionally, HA hydrogel altered glycosylation patterns and modulated key inflammatory and regulatory signaling pathways, resulting in reduced inflammation and regulation of matrix components (Mohd Isa et al., 2018; Mohd et al., 2024) (Figure 4e). The hydrogel also exhibits protective effects by suppressing disc height loss and promoting tissue hydration for structural repair. It upregulates anti-inflammatory cytokines such as Interleukin-10, facilitating disc ECM repair via the transforming growth factor-beta 1 signaling pathway. These mechanisms collectively contribute to its therapeutic potential in addressing disc degeneration-related back pain (Inoue et al., 2021).

Furthermore, recent studies *in vitro*, indicate that crosslinked high molecular weight (Mw) HA hydrogels effectively downregulate inflammatory receptors, including interleukin-1 receptor 1, tumor necrosis factor-alpha, and myeloid differentiation primary response gene 88 *(MyD88).* They also modulate neurotrophins such as nerve growth factor and brain-derived neurotrophic factor (BDNF) in NP cells. This modulation suggests that high Mw HA hydrogels may offer a protective mechanism against inflammation, establishing them as a promising therapeutic strategy for managing intervertebral disc degeneration (Isa et al., 2015).

#### Alginate hydrogel

Alginate, a polysaccharide derived from brown seaweed, has garnered significant interest as a versatile biomaterial due to its ability to form hydrogels that mimic the ECM (Growney Kalaf et al., 2016). Alginate consists of two main fractions: a soluble hydrolyzable fraction and an insoluble fraction, which is resistant to hydrolysis. The insoluble fraction is predominantly composed of molecules rich in either d-mannuronic acid (M) or l-guluronic acid (G) residues, while the soluble fraction contains a higher concentration of alternating MG blocks. The physical properties of alginate are largely determined by the ratio of M and G blocks, which can vary depending on the algal species and the time of harvesting (Chou et al., 2009; Man et al., 2022). Gel formation is mainly driven by G block interactions, which create a stable network (Man et al., 2022).

**FIGURE 5:**
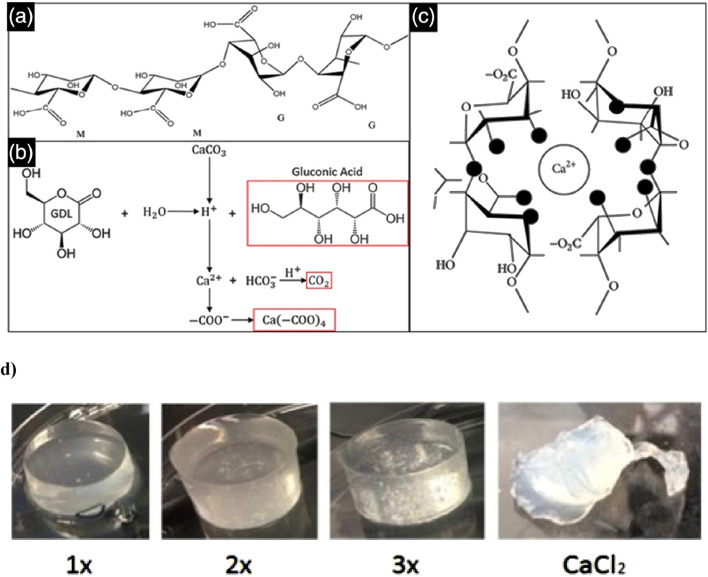
This schematic depicts the alginate crosslinking process. **(a)** Alginate is a polysaccharide composed of β-D-mannuronate (M) and α-L-guluronate (G) residues, with their sequence and ratio influencing gel properties. **(b)** The crosslinking reaction involves GDL as a proton donor and CaCO_3_ as a calcium ion source, generating gluconic acid, carbon dioxide, and a calcium-alginate complex. **(c)** The released Ca^2+^ ions bind to alginate’s carboxyl groups, creating ionic crosslinks that form a stable hydrogel network. Figure adapted from (Gan et al., 2017). **(d)** In gels formulated with a 1:2 ratio of CaCO_3_ to GDL, the 1× CaCO_3_ concentration results in weak crosslinking and an irregular, slightly conical shape due to limited calcium release, leading to lower mechanical stability. The 2× gel demonstrates better uniformity and more complete crosslinking, while the 3× gel exhibits the highest structural integrity, though trapped air bubbles may compromise mechanical strength. In contrast, the CaCl_2_-crosslinked gel undergoes rapid shrinkage and shows poor geometry due to the oversaturation of calcium, resulting in uneven crosslinking and mechanical instability. Figure adapted from (Growney Kalaf et al., 2016).

Alginate can be crosslinked using various methods, each offering distinct advantages and applications. Alginate hydrogels, formed through ionic crosslinking, create supportive matrices that facilitate cell growth and tissue regeneration (Jarrah et al., 2023). Beyond their ECM-like properties, alginate hydrogels also excel in water retention and emulsion stabilization, making them ideal for various tissue engineering applications (Hu et al., 2021). An internal alginate crosslinking method uses calcium carbonate (CaCO_3_) and d-glucono-lactone (GDL), which gradually releases calcium ions to form a hydrogel. This controlled gelation allows for mixing, injection, and *in situ* formation, with tuneable mechanical properties. The method has been used to create injectable alginate scaffolds for osteoblast delivery in tissue engineering (Maxwell et al., 2022) (Figures 5a–c).

In a previous *in vivo* study using a murine subcutaneous pouch model, photo-crosslinked alginate hydrogels showed enhanced gene expression and increased assembly of type II collagen and proteoglycans over 8 weeks (Chou et al., 2009). However, *in vitro* studies have indicated that CaCl_2_-crosslinked alginate gels often lead to cell clumping due to the rapid gelation process, which prevents even distribution of cells throughout the gel. This uneven distribution, combined with an initial decrease in mechanical properties caused by cells metabolizing calcium ions and hindering full crosslinking, presents significant challenges. Nonetheless, mechanical properties tend to stabilize later in culture.

By contrast, alginate hydrogels crosslinked with CaCO_3_ and GDL offer more controlled gelation times (10–30 min) and exhibit stable mechanical properties while demonstrating slow degradation over 28 days. A 1:2 ratio of CaCO_3_ to GDL, using 30 mM and 60 mM concentrations of GDL, has shown exceptional potential for IVD regeneration *in vitro* due to its biocompatibility, high swelling capacity, and stable mechanical properties (Growney Kalaf et al., 2016) (Figure 5d). Significant improvements in swelling capacity *in vitro* have been observed when using a combination of calcium and gallium cations for the cross-linking of alginate hydrogels. The calcium-gallium alginate polymers demonstrated a swelling capacity four times greater than that of gallium alginate alone (Man et al., 2022).

#### Chitosan

Chitosan (CHI) is a naturally derived, non-sulfated GAG commonly used in biomedical applications due to its biodegradability, low toxicity, non-immunogenicity, mucoadhesive properties, and its ability to mimic the ECM in regenerative medicine. It is a linear polymer composed of partly acetylated (1→4)-2-amino-2-deoxy-β-d-glucan units, obtained by isolating chitin through alkaline hydrolysis (Li et al., 2018).

In both *in vitro* and *in vivo* studies, CHI has demonstrated minimal toxicity, with effects largely dependent on its molecular weight and degree of deacetylation. It exhibits low hemolytic activity and is well-tolerated in various animal models, further supporting its safety for biomedical use (Dash et al., 2011). Despite its limitations in mechanical strength and degradation rates for tissue regeneration, CHI’s favorable safety profile supports its potential for further development. To overcome these limitations and enhance CHI’s effectiveness, researchers have explored the creation of composite scaffolds by combining CHI with other functional materials, significantly improving its efficacy in tissue regeneration (Zhao et al., 2023).

For example, *in vitro* studies show that CHI/polyvinyl alcohol (PVA) hydrogels prepared with higher chitosan concentrations demonstrated enhanced mechanical strength, achieving moduli similar to the nucleus pulposus, making them suitable for tissue engineering applications (Enoch et al., 2023). In another study, a novel thermo-sensitive injectable hydrogel was developed using N-hexanoylation of glycol CHI, aimed at treating degenerative disc disease. This hydrogel demonstrated biocompatibility with no cytotoxicity or adverse effects in both *in vitro* and *in vivo* tests, showing promising results in a rat model. Moreover, in an *ex vivo* porcine model, the hydrogel maintained its stability at the defective IVD site for over 28 days, supporting its potential as a long-lasting regenerative therapy (Wang et al., 2024a). Furthermore, an *in vitro* study developed a CHI-gelatin injectable hydrogel supplemented with Link N, which showed significant promise for IVD repair. This composite hydrogel not only improved mechanical properties but also significantly enhanced GAG production in degenerative conditions, highlighting its potential in IVD regeneration applications (Adoungotchodo et al., 2021).

#### Collagen-based hydrogel

Collagen is a family of proteins characterized by a triple-helical structure. Comprises at least 29 distinct types, each playing a crucial role in the ECM of the intervertebral disc. Collagens are abundant in both the AF and NP, where they are key structural components (Xie et al., 2021; Shenoy et al., 2022). Although collagen hydrogels closely emulate the ECM and support excellent cellular interactions, their inherently weak mechanical strength compared to synthetic hydrogels poses a significant limitation, particularly in applications requiring structural integrity and load-bearing capacity (Xu et al., 2022).

**FIGURE 6:**
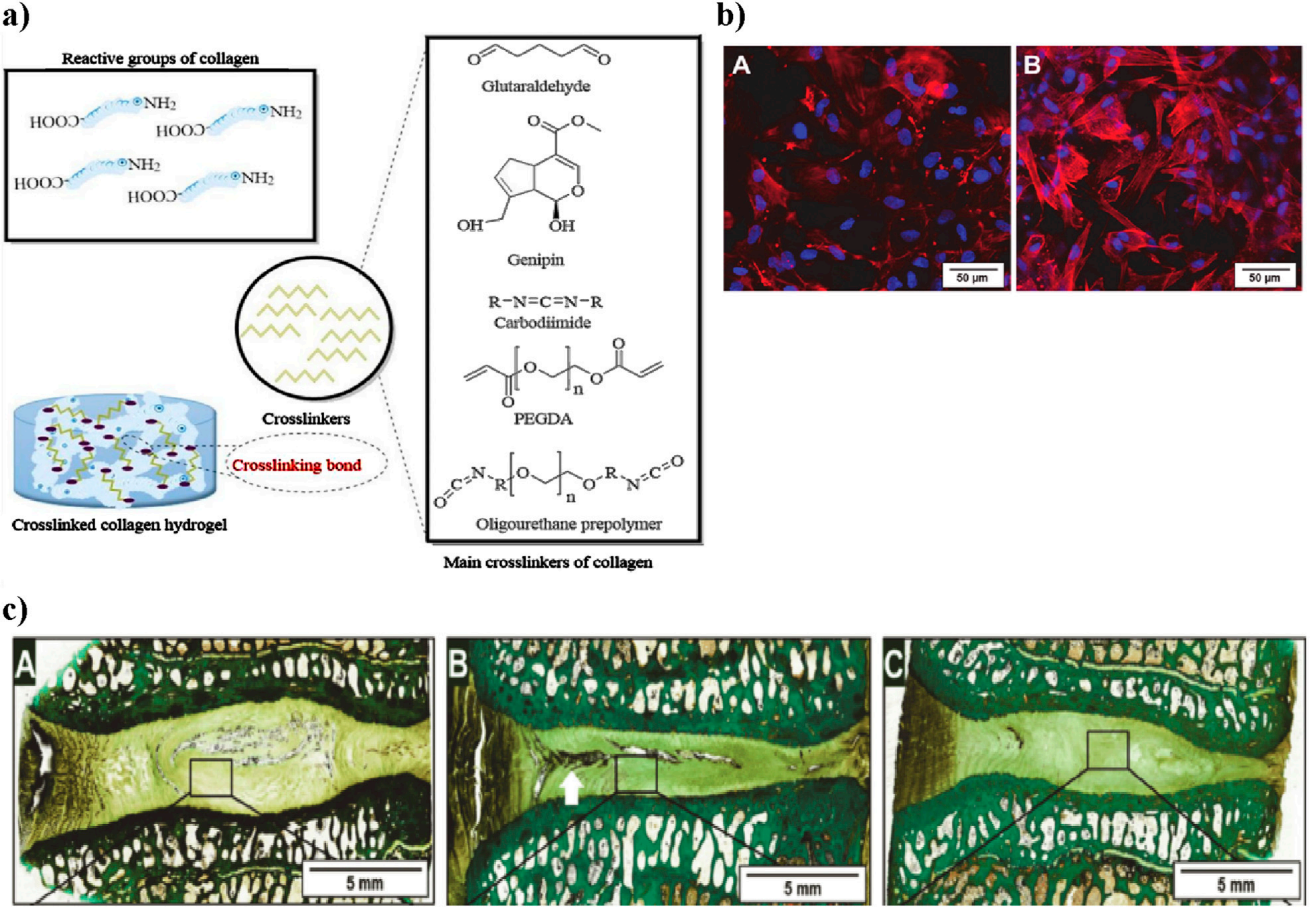
**(a)** Schematic representation of collagen crosslinking. Collagen’s reactive groups (-NH_2_ and -COOH) combine with different chemical crosslinkers to create a stable collagen hydrogel. The main crosslinkers shown include glutaraldehyde, genipin, carbodiimide, PEGDA, and PPU, each influencing mechanical properties and biocompatibility. Figure adapted from (Li et al., 2021). **(b)** Laser scanning microscopy images showing adipose stem cells within the collagen hydrogel. **(a)** After 2 h, cells have spread, and after 36 h, cytoskeletons are formed (red) and nuclei are visible (blue), demonstrating cell vitality. Scale bar: 50 µm. *Adapted from Friedmann et al.* (Wang et al., 2024b). **(c)** Histological sections of intervertebral discs treated with collagen hydrogel loaded with adipose stem cells. Native, untreated disc section showing intact NP structure (A) Damaged disc section post-injury with notable NP degeneration (white arrow indicating damaged region) (B) Collagen hydrogel-treated disc showing partial restoration of NP structure (C) Adapted from Friedmann et al. (Friedmann et al., 2021).

Furthermore, collagen’s poor thermal stability in solution restricts its industrial and biological use. Dialdehyde cellulose is a modified cellulose derivative with aldehyde groups, enabling cross-linking with amino groups (Ding et al., 2018). To overcome these limitations, crosslinking strategies are used to reinforce the fibrous collagen network and improve mechanical support. Chemical crosslinking enhances properties such as mechanical strength, enzymatic degradation resistance, and overall stability by forming covalent bonds between collagen’s amine (-NH_2_) and carboxyl (-COOH) groups and crosslinkers. However, high concentrations of crosslinkers may compromise biocompatibility; hence, minimal effective dosages are preferred. Common crosslinkers include glutaraldehyde (GLU), carbodiimide (EDC), genipin (GEN), polyethylene glycol diacrylate (PEGDA), and polyurethane prepolymers (PPU) (Claudio-Rizo et al., 2019) (Figure 6a).

In this context, a related *in vitro* study by Sargeant et al. demonstrated that an injectable hydrogel system composed of collagen and multi-armed poly (ethylene glycol) (PEG) exhibited tunable mechanical properties, suitable degradation profiles, and the ability to support cellular adhesion and proliferation, highlighting its potential as an effective scaffold for addressing various tissue defects (Sargeant et al., 2012). An *in vitro* study explored the impact of dialdehyde cellulose on collagen cross-linking, showing enhanced hydrothermal stability and resistance to enzymatic degradation (Kanth et al., 2009). The collagen hydrogel showed excellent biocompatibility *in vitro*, as adipose-derived stem cells spread within 2 h and formed clear cytoskeletons after 36 h, indicating strong cell vitality and support for growth (Shenoy et al., 2022) (Figure 6b). An *in vivo* sheep lumbar disc model showed that collagen hydrogels loaded with adipose stem cells. When injected into IVD, resulted in increased disc height over time; however, while this treatment showed improvement compared to damaged discs, it did not fully restore the original height of native discs (Friedmann et al., 2021) (Figure 6c).

When extracted for biomedical use, collagen loses much of its native mechanical strength, thermal stability, and enzymatic resistance, necessitating chemical modification to restore these features (Zhang et al., 2023b). Moreover, direct application can lead to issues such as calcium deposition, thrombogenicity, uncontrollable degradation, and inadequate mechanical performance. To mitigate these problems, functionalization or combination with other biomaterials is often used to enhance the overall performance of collagen hydrogels (Zhao et al., 2023).

#### Fibrin hydrogel

Fibrin is a linear protein formed from the aggregation of fibrinogen in the presence of thrombin, playing a crucial role in blood coagulation and tissue repair. Fibrinogen, primarily produced by hepatocytes, converts into a stable fibrin network that supports healing and is safely degraded without toxic effects, making fibrin valuable in regenerative medicine (Li et al., 2024).

Fibrin-based hydrogels are ideal for tissue engineering due to their excellent biocompatibility, elasticity, non-toxic degradation products, and strong tissue adhesion. They enable high cell seeding efficiency and controllable degradation through protease inhibitors like aprotinin and tranexamic acid. However, in the absence of such modulation, the rapid degradation of fibrin hydrogels can compromise the stability of the 3D cell culture environment, limiting their effectiveness for sustained tissue regeneration (Kotla et al., 2023). As a viscoelastic polymer, fibrin exhibits both elastic and viscous properties, with mechanical characteristics influenced by fiber structure and protofibril assembly. However, challenges such as gel shrinkage and insufficient mechanical strength limit their broader application. To address these issues, additional materials are necessary, and Brinkmann et al. demonstrated that the Tn7 peptide significantly enhances the mechanical properties of fibrin hydrogels (Brinkmann et al., 2023).

In the previous *in vitro* study, AF cells seeded into fibrin gel showed the ability to proliferate and expressed both Type I and Type II collagen; however, they did not express aggrecan or chondroitin-6-sulfotransferase (Colombini et al., 2014; Gruber et al., 2004). Furthermore, Schek et al. found that genipin crosslinked fibrin gels exhibited mechanical properties comparable to native annular tissue, supported the *in vitro* growth of human disc cells, and maintained adhesion to annular tissue under physiological strain, indicating their potential as effective materials for repairing intervertebral disc defects (Schek et al., 2011).

### Synthetic hydrogel

Synthetic hydrogels have emerged as a powerful alternative to natural hydrogels, addressing limitations such as the lack of stability, inadequate mechanical properties, and rapid degradation observed in natural systems (Brinkmann et al., 2023). Examples include polyethylene glycol (PEG), polyvinyl alcohol (PVA), and polyacrylamide (PAM), which provide superior strength and elasticity. Their properties are highly tunable depending on the polymer composition. Compared to natural hydrogels, they demonstrate enhanced water sorption capacity, stability, and gel strength, primarily due to their chemically crosslinked structure (Tsou et al., 2016; Popa, 2023). However biomedical applications of synthetic hydrogels were initially hindered by issues like toxic unreacted monomers, low biodegradability, and challenges in hydrogel formation under physiological conditions (Nikolić et al., 2018). Concerns about biocompatibility, toxic degradation products, and their limited ability to support cell attachment also posed obstacles (Brinkmann et al., 2023). For example, glutaraldehyde, a common cross-linking agent, may cause mild cytotoxicity depending on its concentration and molar ratios (Blinova et al., 2024).

#### Polyethylene glycol

PEG polymers, widely used as excipients in pharmaceuticals, consist of repeated ethylene glycol units [-(CH_2_CH_2_O)_n_ (Pham Le Khanh et al., 2022; D’souza and Shegokar, 2016). PEG varies in Mw from 200 to 10,000,000 g/mol. Their physical state changes with Mw: low Mw PEGs (200–700) are liquid, medium Mw PEGs (800–2000) are semisolid, and high Mw PEGs (3,000+) are solid (Pham Le Khanh et al., 2022). Its derivatives, such as polyethylene glycol methacrylate (PEGMA), polyethylene glycol dimethacrylate (PEGDMA), and PEGDA, are commonly applied in drug delivery systems, tissue engineering, and cell encapsulation (Gdansk University of Technology et al., 2010). While PEG is generally well-tolerated, some studies have reported systemic reactions, such as muscle weakness, nausea, pain, pruritus, and sensory loss (Argentieri et al., 2025).

**FIGURE 7:**
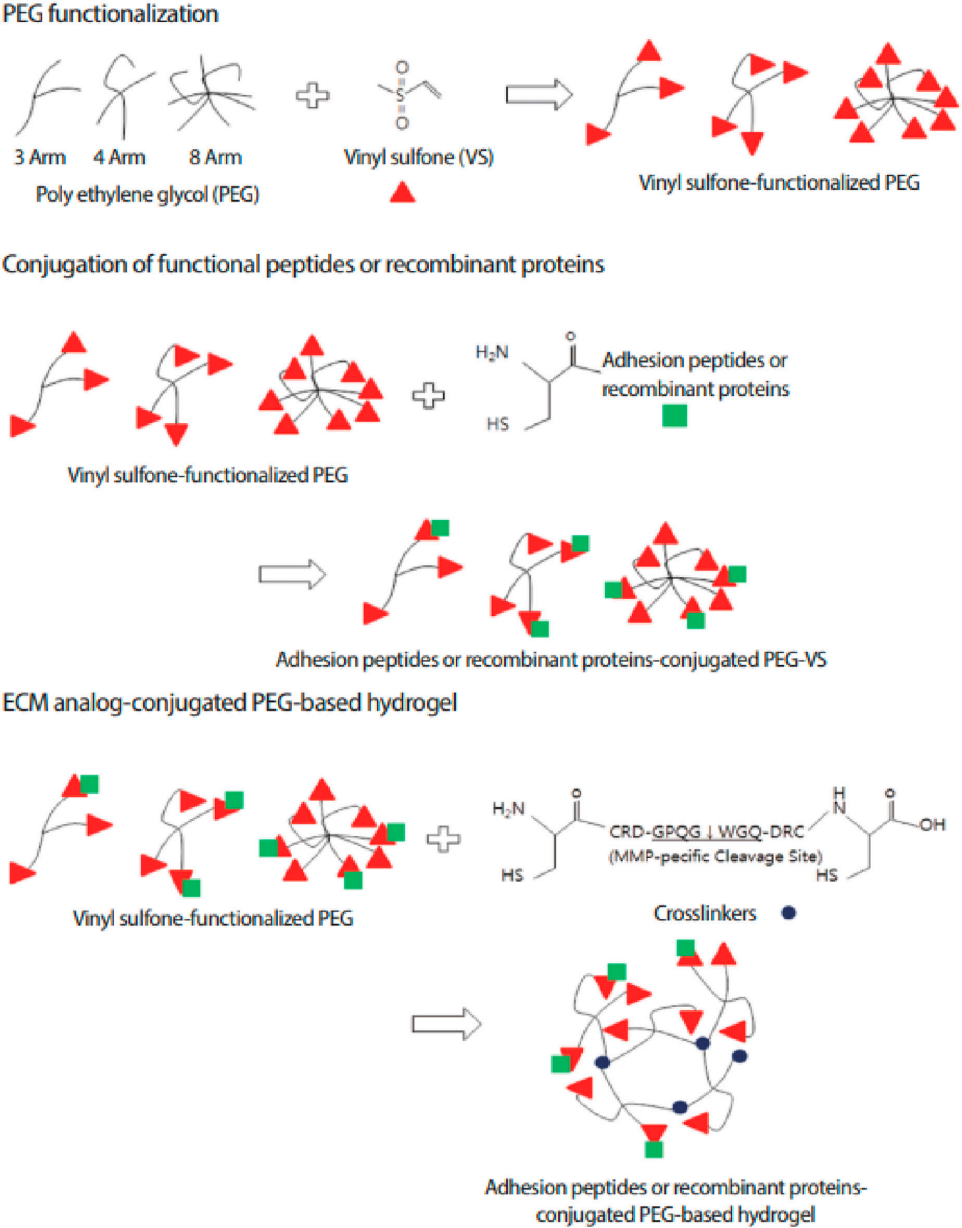
Schematic representation of ECM analogue-conjugated PEG-based hydrogel formation. Vinyl sulfone (VS)-functionalized PEG is crosslinked using MMP-sensitive peptides, allowing controlled degradation in response to enzymatic activity. This strategy enhances bioactivity by enabling cell adhesion through conjugated peptides or recombinant proteins. Figure adapted from (Lim et al., 2013).

PEG-based hydrogels are widely used in tissue engineering due to their non-immunogenicity, high water content, resistance to protein adsorption, tuneable mechanics, and ease of functionalization for peptide and protein coupling (Jeong et al., 2014). PEG-based hydrogels can be chemically cross-linked for controlled drug release and tissue regeneration, responding to stimuli like temperature, pH, and molecular interactions (Gdansk University of Technology et al., 2010). A common approach to functionalizing PEG hydrogels involves modifying PEG with vinyl sulfone (VS) groups (Figure 7). VS-functionalized PEG hydrogels incorporate dicysteine-containing peptides with MMP-specific cleavage sites, enabling controlled degradation in response to cellular activity. The thiol (-SH) groups of cysteine conjugate with VS groups via a Michael-type addition reaction, forming a stable yet bioresponsive crosslinked network in a cell-friendly environment. These advances have led to the development of PEG-based artificial niches for regulating stem cell function and activity (Lim et al., 2013).

Although PEG-based hydrogels match the biomechanical properties of articular cartilage—such as compression, tensile strength, and hydrostatic swelling—they are seldom used alone in regenerative medicine due to their inherent cell-repellent nature, which stems from poor protein adsorption (Jeong et al., 2014; Guarnieri et al., 2010). This non-cell adhesive behavior limits cell attachment and interaction, posing challenges for effective tissue engineering (Jeong et al., 2014). For example, an *in vitro* study using an injectable hydrogel of PEG and HA crosslinked with Horse Radish Peroxidase (HRP), demonstrated promising properties for intervertebral disc regeneration, including ideal swelling, increased degradation at lower HRP concentrations, and over 90% cell viability, indicating good biocompatibility (Kwarta et al., 2017). Different PEG shapes can be created by using various initiators (e.g., hexa-glycerin for tri-PEG) or by linking linear PEGs (Pasut et al., 2016). Different shapes of PEG can be created using various initiators, such as hexa-glycerin for tri-PEG, or by linking linear PEGs. Recent advancements include multi-functional PEGs like 4S-Star PEG, which act as injectable crosslinkers to improve scaffold stability by binding to free amine groups (Mohd Isa et al., 2023; Duan et al., 2021). An *in vitro* study found that a hydrogel made from type II collagen and HA crosslinked with 4S-Star PEG enhances thermal stability and osteogenic activity (Mohd Isa et al., 2023).

#### Polyvinyl alcohol (PVA)

PVA is a water-soluble polymer derived from polyvinyl acetate hydrolysis. Its polar hydroxyl groups facilitate strong hydrogen bonding, leading to excellent film formation, solubility, emulsification, and adhesion. This versatility makes PVA highly suitable for biomedical applications (Ma et al., 2017). PVA hydrogels have garnered significant interest because of their low toxicity, excellent water absorption, strong mechanical properties, and favorable biocompatibility (Wang M. et al., 2021). Traditionally, PVA hydrogels are prepared using a repeated freeze-thaw approach. However, recent research has introduced a novel mixed solvent physical cross-linking method. This new approach creates hydrogels with high water content, outstanding re-swelling rates, superior melting temperature, and strong mechanical integrity. Notably, these hydrogels maintain structural stability across a wide temperature range (20°C–65°C), making them ideal for tissue engineering applications (Ma et al., 2017).

Compared to PVA hydrogels crosslinked with synthetic materials, which often lack bioactivity, those crosslinked with natural materials offer enhanced compatibility for cell culture and tissue growth (Subagio et al., 2023). Furthermore, PVA hydrogel exhibits biomechanical characteristics similar to those of the native NP, particularly at 15 wt% and 20 wt% concentrations. Increasing PVA content enhanced the Young’s modulus while decreasing permeability, aligning the hydrogel’s properties with those of the NP. Dynamic motion analysis showed that 20 wt% PVA hydrogel provided a comparable range of motion and facet joint forces to the natural lumbar disc, making it the optimal choice for NP replacement and promoting structural stability (Subagio et al., 2023; Heo and Park, 2022).

#### Polyacrylamide

Polyacrylamide (PAM) is synthesized through *chain-growth polymerization*, where individual acrylamide monomers connect in a sequence to form long polymer chains. PAM hydrogels are formed by crosslinking acrylamide monomers with a crosslinking agent, typically N,N′-methylene bis (acrylamide) (Bis), and offer flexible modification options, making them ideal for studying ECM effects on cell behavior (Liu et al., 2019). PAM hydrogel is widely applied in tissue engineering for its hydrophilicity, excellent swelling capacity, and non-toxic nature (Jafari et al., 2022; Fang et al., 2019). It combines narrow hysteresis with impressive toughness, making it an ideal material for a range of biomedical applications (Shenoy et al., 2022). Polyacrylamide’s inertness allows scientists to customize cell attachment by incorporating specific molecules, but it also complicates the process of bonding these molecules effectively (Gribova et al., 2011).

It is important to note that pure polyacrylamide gels are inherently brittle, which can restrict their mechanical performance and low biodegradable (Kundu and Kundu, 2012; Autónoma de Coahuila et al., 2023). To enhance their properties, the hydrogel needs to combine with other materials. Hydrogels made from a combination of gelatin and polyacrylamide have demonstrated *in vitro* tunable Young’s moduli ranging from 5 to 35 kPa and swelling ratios between 947% and 1,654%. Increasing the AAm/Bis ratio enhances swelling capacity due to a looser crosslinked structure and allows for precise adjustments in Young’s modulus by modulating crosslinking density. These composite hydrogels maintain structural integrity under cyclic loading and exhibit excellent biocompatibility, promoting cell attachment (Jafari et al., 2022).

#### Polyurethane

Polyurethane (PU) is synthesized from polyols and diisocyanates, often with a crosslinker (Gribova et al., 2011; Kundu and Kundu, 2012). Different polyols such as polyester, polyether, polycarbonate, and polycaprolactone affect PU properties: high-molecular-weight polyols lead to flexibility, while low-Mw polyols create rigidity. In this process, isocyanates (–N=C=O) react with nucleophiles, with aromatic isocyanates being more reactive than aliphatic ones, making them suitable for rigid PUs.

PUs exhibit low cytotoxicity, high oxygen permeability, strong thrombo-resistance, excellent biocompatibility, biodegradability, and tissue-like mechanical properties (Naureen et al., 2021). Polycarbonate-based PU scaffolds, in particular, provide additional benefits such as strength, elasticity, and support for cell adhesion, ECM production, and alignment of native IVD cells (Turner et al., 2014; Agnol et al., 2018). Recent advancements have introduced a bi-phasic PU scaffold with a hydrophilic core for rapid swelling and an electrospun envelope for structural support and cell attachment. This design enables minimally invasive delivery and supports rapid disc height restoration in NP applications (Li et al., 2016). Additionally, tensile forces on PU scaffolds have been shown to enhance AF cell alignment and matrix production, making optimized PU scaffolds promising for both NP and AF engineering (Turner et al., 2014).

### Composite hydrogel

Native ECMs in living tissues act as finely tuned composite hydrogels, where fibrous networks like collagen are embedded within hydrated polysaccharides and glycosylated protein matrices. Inspired by this structure, synthetic composite hydrogels combining multiple materials with synergistic properties have been developed to enhance cell support in 3D models. While traditional natural or synthetic polymer-based hydrogels lack the ability to fully replicate the native ECM, these composite hydrogels offer a promising approach to overcome these limitations (Zhao et al., 2020). Most hydrogels lack the mechanical strength of natural tissues, and improving this is a challenge, which can be addressed by using hydrogel composite systems that combine multiple strengths and functions (Tajik et al., 2023).

For example, Agarose, a polysaccharide from red algae, forms strong, reversible gels without additives. It's widely used in 3D cell culture due to its non-toxicity, affordability, and large pore size. However, it has limited cell invasion, which can be improved with agarose/collagen composites (Shin et al., 2016). The bioengineering of composite hydrogels for intervertebral disc replacement is emerging, with research aiming to optimize polymer performance (Desai et al., 2024a). However, some limitations still remain, such as unknown long-term biocompatibility, potential nanotoxicity, and concerns about hydrogel extrusion from the IVD space under mechanical loads (Devi and Gaba, 2019; Desai et al., 2024b; Reitmaier et al., 2014). An *in vivo* sheep model, methacrylated gellan gum hydrogel failed to restore nucleus pulposus function, likely due to extrusion from the intervertebral disc space (Reitmaier et al., 2014).

Nanocomposite hydrogels, formed by integrating nanoscale fillers into a hydrogel matrix, enhance mechanical performance and efficiently transmit mechanical stimuli to cells (Xing and Tang, 2022; Pina et al., 2015). The nanosized fillers transform the polymer matrix’s physical properties, enabling superior biomaterials beyond what individual components can achieve (Pina et al., 2015). Nanomaterials can be made from polymers, ceramics, metals, or carbon-based structures, typically ranging in size from 1 to 100 nm (Chen et al., 2024). To achieve a uniform distribution of nanoparticles within these hydrogels, researchers have developed five primary strategies: (Uysal et al., 2019): forming the hydrogel directly in a nanoparticle suspension, (World health organization, 2023), embedding nanoparticles into the hydrogel matrix after gelation, (Wu et al., 2020), synthesizing reactive nanoparticles within a preformed gel, (Basatvat et al., 2023), employing nanoparticles as cross-linkers to stabilize the hydrogel, and (Baumgartner et al., 2021) forming gels with nanoparticles, polymers, and distinct gelator molecules (Thoniyot et al., 2015).

Recent advancements in nanotechnology underscore the potential of nanocomposite hydrogels for treating degenerative joint diseases (Chen et al., 2024). A practical example is CHI hydrogels reinforced with nanofibrillated cellulose for AF repair. This nanofibrillated cellulose-CHI composite enhances structural strength, supports IVD biomechanics, and prevents nucleus protrusion. *Ex vivo* pig spine studies highlight its regenerative potential for AF defects (Doench et al., 2019). Similarly an *in vivo* study using a rat model, calcium alginate nanocomposite hydrogels have shown promise in drug delivery applications, offering antioxidative, anti-inflammatory, and cell migration benefits, positioning them as innovative solutions for intervertebral disc repair (Zheng et al., 2023).

## Critical comparison of different hydrogels for IVD regeneration

As shown in Table 2, a variety of biomaterials have been explored for IVD regeneration, including both natural and synthetic hydrogels. These materials have been evaluated *in vitro, ex vivo,* and *in vivo*. Each hydrogel type presents unique advantages and challenges depending on the therapeutic goal. For example, In a rabbit *in vivo* model, HA-based hydrogels reduced inflammation, modulated pain, and limited disc height loss in the lumber region by upregulating IL-10 and promoting ECM repair via the TGF-β1 pathway (Inoue et al., 2021). Gelatin-based BIOGEL hydrogels also demonstrate promise by closely mimicking the native disc’s biomechanical environment. Their rapid crosslinking and mechanical integrity, coupled with TGF-β incorporation, enhanced matrix repair, modulated cytokines, and alleviated pain in animal models (Luo et al., 2022).

In contrast, PEG-based hydrogels are highly tunable in terms of mechanical stiffness (ranging from 0.2 to 4.5 kPa) and allow for controlled delivery of therapeutic agents, such as decellularized notochordal matrix (dNCM), over extended periods. *In vitro*, PEG hydrogels support cell viability; *in vitro*, they improve disc height index and endplate organization. However, encapsulated notochordal cells in these hydrogels tend to lose metabolic activity and phenotypic expression over time, indicating the need for further refinement (Schmitz et al., 2023). Additionally, PEG hydrogels require biomechanical optimization for better integration under physiological loading conditions (Desai et al., 2024b).

Other materials, such as chitosan-based hydrogels, offer mechanical properties comparable to the native NP. When combined with gelatin and the Link N peptide, chitosan hydrogels significantly enhanced GAG production, showing a 4.7-fold increase in NP cells cultured in a degenerative environment. This finding suggests potential for restoring disc function during early-stage degeneration (Adoungotchodo et al., 2021). Similarly, Si-HPMC hydrogels support cell viability through efficient nutrient diffusion. However, their neutral charge limits cell adhesion and proliferation, which may reduce their effectiveness in applications that require extensive cell-matrix interactions (Nativel et al., 2018).

However, despite their potential, hydrogels—whether synthetic, natural, or composite—face several challenges that must be addressed for successful clinical translation. Natural hydrogels often suffer from weak mechanical strength and stability, making them prone to rupture or collapse under stress, such as compression or tension, when implanted (Tajik et al., 2023; Ho et al., 2022). Even synthetic hydrogels offer controllable molecular weight, adjustable mechanical properties, and affinity for carriers like drugs and cells, but their challenging degradation, biocompatibility concerns, toxic byproducts, and limited cell attachment hinder their clinical use (Blinova et al., 2024). While composite hydrogel offers numerous benefits in biomedical applications also presents several limitations.

## Translational challenges in extracellular matrix hydrogel-based intervertebral disc regeneration

Several hydrogel-based systems have received regulatory approvals from the U.S. Food and Drug Administration (FDA) or Conformité Européenne (CE) for clinical applications in areas such as wound healing, drug delivery, and soft tissue repair. However, in the context of IVD treatment, despite extensive preclinical progress, only a limited number of hydrogel systems have advanced to clinical evaluation under regulatory oversight (Mohd Isa et al., 2022).

ReGelTec Inc. has developed HYDRAFIL, a thermoresponsive injectable hydrogel composed of a proprietary polymer, designed to mimic the native nucleus pulposus, for degenerative disc disease that is currently under investigation in an FDA-regulated clinical trial. Although still classified as an unapproved device, its evaluation under FDA supervision represents a significant step toward clinical translation (ReGelTec and Inc, 2025a). The transition from bench to bedside in IVD therapy is complex and influenced by numerous factors. Hydrogels must withstand approximately one million spinal loading cycles annually, yet few achieve this in long-term mechanical testing (Tang et al., 2020). Additionally, ECM-based and other injectable hydrogels face multiple translational hurdles such as inadequate cross-linking, low mechanical strength, and instability under physiological conditions including moisture, pH, and temperature variations, necessitating the development of more responsive and durable designs (Desai et al., 2024a; Kaur and Murphy, 2023).

Traditional covalent hydrogels often require invasive implantation and lack compatibility with emerging manufacturing techniques like 3D printing, whereas injectable formulations must be optimized for needle gauge, flow rate, and compatibility with therapeutic cargo (Correa et al., 2021). Challenges also include the need for photoreversible hydrogels that respond to longer wavelengths with higher quantum efficiency, as well as ensuring biocompatibility and minimizing potential damage from photothermal reactions on biological molecules (Liu et al., 2023a). Improper cross-linking can result in mechanical mismatches with native IVD tissue, increasing the risk of deformation and rupture in load-bearing environments (Baumgartner et al., 2021; Liu et al., 2023b), while weak and flexible cross-links limit shape adaptability and resilience (Ning et al., 2022). Furthermore, pH-responsive hydrogels may be ineffective in the IVD’s stable microenvironment, and issues like premature gelation within syringes or needles can impede delivery (Parhi, 2017; Naficy et al., 2024). Injection-induced tissue damage may also compromise surrounding IVD structures, reducing treatment efficacy (Kaur and Murphy, 2023). While various hydrogel products have secured FDA or CE approval in other medical fields, those designed for IVD therapy must meet significantly more stringent requirements related to biomechanics, biocompatibility, and scalable manufacturing to achieve successful clinical translation (Gao et al., 2021). Additionally, although hydrogels can serve as promising delivery vehicles for therapeutic agents in IVD regeneration, their hydrophilic nature makes the sustained delivery of hydrophobic drugs—often needed to control inflammation or pain, particularly challenging (Lu et al., 2024).

## Conclusions and future perspectives

Emerging research highlights hydrogels as a promising therapy for IVDD, offering both symptom relief and regenerative capabilities by mimicking the ECM and providing structural support (Desai et al., 2024a; Geckil et al., 2010). These biomaterials promote cellular regeneration and restore disc functionality, marking a shift toward biologically driven treatments for chronic LPB.

Advancements in hydrogel design—such as tailored degradation rates, controlled drug release, and patient-specific formulations—may enable more personalized therapies. Despite challenges like optimizing stability, ensuring biocompatibility, and navigating regulatory pathways, interdisciplinary collaboration could position hydrogel-based ECM mimics as versatile tools in tissue engineering. By pushing the boundaries of biomimetic technology, these innovations hold promise for durable treatments that improve patient outcomes globally.
